# Comparative genomics of the bacterial genus *Listeria*: Genome evolution is characterized by limited gene acquisition and limited gene loss

**DOI:** 10.1186/1471-2164-11-688

**Published:** 2010-12-02

**Authors:** Henk C den Bakker, Craig A Cummings, Vania Ferreira, Paolo Vatta, Renato H Orsi, Lovorka Degoricija, Melissa Barker, Olga Petrauskene, Manohar R Furtado, Martin Wiedmann

**Affiliations:** 1 Department of Food Science, Cornell University, Ithaca NY, 14853, USA; 2 Life Technologies Corporation, 850 Lincoln Centre Drive, Foster City, CA 94404, USA

## Abstract

**Background:**

The bacterial genus *Listeria *contains pathogenic and non-pathogenic species, including the pathogens *L. monocytogenes *and *L. ivanovii*, both of which carry homologous virulence gene clusters such as the *prfA *cluster and clusters of internalin genes. Initial evidence for multiple deletions of the *prfA *cluster during the evolution of *Listeria *indicates that this genus provides an interesting model for studying the evolution of virulence and also presents practical challenges with regard to definition of pathogenic strains.

**Results:**

To better understand genome evolution and evolution of virulence characteristics in *Listeria*, we used a next generation sequencing approach to generate draft genomes for seven strains representing *Listeria *species or clades for which genome sequences were not available. Comparative analyses of these draft genomes and six publicly available genomes, which together represent the main *Listeria *species, showed evidence for (i) a pangenome with 2,032 core and 2,918 accessory genes identified to date, (ii) a critical role of gene loss events in transition of *Listeria *species from facultative pathogen to saprotroph, even though a consistent pattern of gene loss seemed to be absent, and a number of isolates representing non-pathogenic species still carried some virulence associated genes, and (iii) divergence of modern pathogenic and non-pathogenic *Listeria *species and strains, most likely circa 47 million years ago, from a pathogenic common ancestor that contained key virulence genes.

**Conclusions:**

Genome evolution in *Listeria *involved limited gene loss and acquisition as supported by (i) a relatively high coverage of the predicted pan-genome by the observed pan-genome, (ii) conserved genome size (between 2.8 and 3.2 Mb), and (iii) a highly syntenic genome. Limited gene loss in *Listeria *did include loss of virulence associated genes, likely associated with multiple transitions to a saprotrophic lifestyle. The genus *Listeria *thus provides an example of a group of bacteria that appears to evolve through a loss of virulence rather than acquisition of virulence characteristics. While *Listeria *includes a number of species-like clades, many of these putative species include clades or strains with atypical virulence associated characteristics. This information will allow for the development of genetic and genomic criteria for pathogenic strains, including development of assays that specifically detect pathogenic *Listeria *strains.

## Background

The eight recognized species within the genus *Listeria *include *L. monocytogenes*, *L. innocua*, *L. welshimeri*, *L. seeligeri*, *L. ivanovii*, *L. grayi*, *L. marthii *[1] and *L. rocourtiae *[2], the latter two were described in 2009. *L. grayi *is only distantly related to the other *Listeria *species [1,3] and has been proposed to represent a different genus, *Murraya *[4]. *L. monocytogenes *and *L. ivanovii *are pathogens of warm-blooded hosts. *L monocytogenes *causes a severe foodborne disease in humans as well as invasive infections in a number of other warm-blooded host species, particularly ruminants. *L. ivanovii *predominantly causes infections in ruminants, but has also been associated with rare infections in humans [5,6]; this species is considered to have a narrower host range than *L. monocytogenes *[7]. Interestingly, each of these two pathogenic *Listeria *species is closely related to non-pathogenic species; *L. monocytogenes *is closely related to *L. innocua *and *L. marthii *[1], and *L. ivanovii *is closely related to *L. seeligeri *[3,8], which is non-pathogenic even though many isolates contain a homologue of the main *Listeria *virulence gene cluster.

Genome sequencing efforts for *Listeria *have, so far, largely focused on *L. monocytogenes*; as of August 15, 2010, 25 *L. monocytogenes *genome sequences are publicly accessible in standard sequence databases (GenBank; EMBL). Most of these *L. monocytogenes *genome sequences represent strains classified into the two most common *L. monocytogenes *phylogenetic lineages [9] including lineage I (e.g. strains F2365, H7858 [10] ) and lineage II (e.g. strains EGD-e [11], 08-5578 and 08-5923 [12]). The other two *L. monocytogenes *phylogenetic lineages (III and IV) are only represented by 3 genome sequences (i.e., strains HCC23 [Genbank acc. CP001175], FSL J2-071 [Genbank acc. ARN00000000], and FSL J1-208 [Genbank acc. AARL00000000]). Publicly available genome sequences for other *Listeria *species include those for *L. innocua *CLIP11262 [11] and *L. welshimeri *SLCC5334 [13] as well as recently released the genome sequences for *L. seeligeri *SLCC3954 [14] and *L. grayii DSM 20601 *(Genbank acc. ACCR00000000). Knowledge of the genomic content of non-pathogenic relatives of pathogenic species is necessary though to understand the evolution of virulence associated genes and to facilitate identification of putative virulence genes [15].

The main *Listeria *virulence gene cluster (also known as the *prfA *virulence cluster or the *Listeria *pathogenicity island [LiPI]) encodes a number of proteins that are necessary for intracellular survival and motility [16,17]. Specific functions encoded in this cluster include hemolysin, two phospholipases and a metalloprotease (encoded by *hly*, *plcA*, *plcB*, and *mpl*), which all contribute to escape from host cell vacuoles, an actin polymerizing protein (encoded by *actA*), and a global regulator of virulence gene transcription (encoded by *prfA*). Members of the internalin protein family, which are cell wall anchored or secreted proteins that are characterized by the presence of leucine rich repeats, are also associated with virulence in different *Listeria *strains. While a considerable number of genes encoding internalins have been reported in pathogenic and non-pathogenic *Listeria *[11,18,19], clear virulence related functions have only been assigned to a few internalins, including *inlA *and *inlB*, which encode proteins required for invasion of different cells types, including human intestinal epithelial cells [20], and *inlC *[21]. A number of atypical *Listeria *strains and lineages have been reported [22-24], including several putative evolutionary intermediates, which are characterized by unique virulence gene presence/absence patterns. For example, while the non-pathogenic *L. innocua *is typically non-hemolytic and lacks the *prfA *cluster, a small number of strains that contain the *prfA *cluster as well as *inlA *have been reported [22,23]. Also, non-hemolytic *L. seeligeri *strains that lack the *prfA *cluster have been reported [24]; even though many *L. seeligeri *contain the *prfA *cluster, isolates in this species are avirulent in typically studied mammalian hosts [25].

Based on the observations outlined above, we propose that the genus *Listeria *represents an outstanding model system for studying the evolution of pathogenicity and the transition between pathogenic and saprotrophic lifestyles using a comparative genomics approach. We thus performed genome sequencing of (i) isolates representing *Listeria *species (except for *L. grayi*) for which no genome sequences are publicly available and (ii) atypical strains of *Listeria *species for which genome sequences of typical strains were already available. While *L. marthii *was included in our genome sequencing efforts, *L. rocourtiae *was not as this new species was described after completion of the work reported here.

## Methods

### Selection of isolates for genome sequencing

The isolates sequenced in this study (Table 1) were selected to (i) cover the full phylogenetic diversity of the genus *Listeria *(except for *L. grayii*) [9], and to (ii) represent atypical phenotypes (e.g., hemolytic *L. innocua*, non-hemolytic *L. seeligeri*) of some non-pathogenic species. *L. seeligeri *FSL N1-067 was selected as a typical hemolytic strain, while *L. seeligeri *FSL S4-171 represents an atypical non-hemolytic strain of the same species. *L. marthii *FSL S4-120 is the type strain of *L. marthii *and has recently been shown to represent the most closely related species to *L. monocytogenes*, however it is not pathogenic. *L. ivanovii *subsp. *londoniensis *FSL F6-596 (ATCC 49954) represents the type strain of this subspecies and represents the second pathogenic species in *Listeria*. *L. monocytogenes *FSL F2-208 represents lineage IIIC, a distinct lineage within *L. monocytogenes *that has not been sequenced before. *L. innocua *FSL J1-023 was sequenced because this strain represents a atypical hemolytic *L. innocua *isolate, while FSL S4-378 was sequenced as an additional typical non-hemolytic strain of *L. innocua *to further assess intraspecific genomic variation within *L. innocua*. Hemolytic activity for all strains was previously tested [26].

**Table 1:** Strains used for comparative genomic analysis

**Strain designation**^**a**^	**Source, geographic origin, lineage**^**b**^	**Genome size (Mbp)**^**C**^	Hemolytic activity	**Pathogen**^**d**^	**Genbank acc. number or Genome project ID**^**e**^
***L. monocytogenes***					
F2365^†^	food, listeriosis outbreak, CA, USA, 1985, lineage I	2.91	+	+	AE017262
EGD-e^†^	lab strain derived from isolate of rabbit, England, 1924, lineage II	2.94	+	+	AL591824
FSL F2-208*	blood, human listeriosis case, USA, 1999, lineage IIIC	3.20	+	+	ADXE00000000*
HCC23^†^	naturally avirulent serotype 4a strain from catfish, USA, lineage IIIA	2.98	+	-	CP001175
CLIP80459^†^	human epidemic, France, 1999, lineage I	2.91	+	+	FM242711
***L. marthii***					
FSL S4-120*	soil, forest, NY, USA, 2001	2.87	-	-	ADXF00000000*
***L. innocua***					
CLIP11262^†^	food, Morocco	3.01	-	-	AL592102
FSL S4-378*	puddle of water, NY, USA, 2002	3.09	-	-	ADXG00000000*
FSL J1-023*	obtained from Qualicon, exact origin unknown	2.91	+	-	ADXH00000000*
***L welshimeri***					
SLCC5334^†^	decaying vegetation, USA	2.81	-	-	AM263198
***L. ivanovii***					
FSL F6-596*	food, France	3.10	+	+	ADXI00000000*
***L. seeligeri***					
FSL N1-067*	food processing plant, NY, USA	3.09	+	-	ADXJ00000000*
FSL S4-171*	urban environment, NY, USA, 2001	2.89	-	-	ADXK00000000*

^a ^A † indicates strains for which the complete genome sequence is publicly available, an * indicates strains that were sequenced as part of the study reported here. The *L. ivanovii *strain sequenced here is the type strain of *L. ivanovii *subsp *londoniensis *(ATCC 49954). *L. monocytogenes *FSL R2-574, a duplicate of *L. monocytogenes *F2365, was sequenced to assess the performance of the sequence and genome assembly methods.

^b ^For *L. monocytogenes *the intraspecific phylogenetic lineage to which the isolates belong is indicated.

^c ^Sizes of newly sequenced genomes were derived from the sum of the length of all contigs from the *de novo *assembly.

^d ^All *L. monocytogenes *strains were classified as pathogens based on their involvement in clinical cases and/or their virulence in mouse studies (i.e., EGD-e [86], F2365 [87]) except for *L. monocytogenes *HCC23, which has been shown to be non-pathogenic [78]. The hemolytic *L. innocua *FSL J1-023 was considered non-pathogenic based on its avirulence in a mouse infection experiment [22]. All non-hemolytic strains were considered avirulent as they lack the *prfA *virulence cluster. Avirulence for some strains was also supported by animal experiments (i.e., *L. welshimeri *[77]; *L. innocua *CLIP11262 [21]) or tissue culture data (i.e., *L. marthii *[1]). The hemolytic *L. seeligeri *strain was considered avirulent as it shows low invasion efficiencies for Caco-2 cells (comparable to avirulent *L. innocua*; see Figure 5) and as other hemolytic *L. seeligeri *strains have been shown to be avirulent in mouse infection experiments [77].

^e ^Accession numbers marked with an asterisk have been deposited as Whole Genome Shotgun projects at DDBJ/EMBL/GenBank under the accession xxxx00000000. The version described in this paper is the first version, xxxx01000000.

### Genome sequencing and assembly

Genomic DNA was isolated using the UltraClean Microbial DNA Isolation Kit (MO BIO Laboratories, Carlsbad, CA) according to manufacturer's instructions. *Listeria *genomes were sequenced using the SOLiD™ 3 system (Applied Biosystems, Foster City, CA) following manufacturer's protocols. Mate-paired libraries with approximately 1.5 kb inserts were constructed from 20 μg of genomic DNA, and deposited on one quarter of a flow cell. Twenty-five base reads were obtained from each of the F3 and R3 tags, with 27 million to 57 million reads obtained for each of the genomes. After correcting errors in colorspace reads using a modified version of the spectral alignment tools from the EULER-USR package [27], *de novo *assembly was performed using the SOLiD™ System *de novo *Accessory Tools (http://solidsoftwaretools.com/gf/project/denovo/), which employs the Velvet assembly engine [28].

In order to identify likely misassemblies, scaffolds were aligned using MUMmer [29] to the most closely related reference genome available at the time of the analysis: *L. monocytogenes *scaffolds were aligned to *L. monocytogenes *F2365, *L. innocua *and *L. marthii *scaffolds were aligned to *L. innocua *Clip11262, and *L. ivanovii *and *L. seeligeri *scaffolds were aligned to *L. welshimeri *SLCC5334. Scaffolds were broken at points where non-contiguous regions of the reference genome were juxtaposed, and then ordered such that they were syntenic with the reference genome. All scaffolds were then concatenated into a single pseudogenome, separated by the sequence NNNNNCACACACTTAATTAATTAAGTGTGTGNNNNN, which puts stop codons in all six reading frames. Scaffolds that did not match the reference genome were concatenated in arbitrary order at the end of the pseudogenome.

The genome sequences of the seven newly sequenced strains have been deposited to GenBank as whole genome shotgun projects (see table 1 for accession numbers).

### Genome annotation and whole genome alignments

Concatenated pseudogenome sequences were run through JCVI's prokaryotic annotation pipeline (http://www.jcvi.org/cms/research/projects/annotation-service/), which includes gene finding with Glimmer, Blast-extend-repraze (BER) searches to extend ORF finding beyond premature stop codons, HMM searches against Pfam [30] and TIGRFAM [31], TMHMM searches, SignalP predictions, and automatic annotations from AutoAnnotate. The manual annotation tool Manatee (downloaded from http://manatee.sourceforge.net) was used to manually review the output and aid in genome annotation and gene identification. Whole genome alignments were created in Mauve 2.3.0 [32] using the Progressive Mauve algorithm.

### Orthologue analyses

Initially, orthologues found in six publicly available complete *Listeria *genomes (see Table 1) were identified using BLASTCLUST [33]. This analysis was limited to these six complete genomes to avoid possible problems with (sometimes incomplete) fragmented draft sequences such as the genome sequences available in the Broad Institute *L. monocytogenes *database (http://www.broadinstitute.org/annotation/genome/listeria_group/MultiHome.html), at the time of this analysis. Only ORFs having at least 225 nt (encoding 75 amino acids [aa]) were considered in this analysis as smaller ORFs were not annotated consistently among the genomes. A set of 3,668 unique ORFs, found among these six complete genomes by requiring 75% aa identity over at least 80%, was used to identify orthologues of these ORFs in the new genome assemblies (using TBLASTN queries with a single member of each cluster and requiring at least 65% aa identity). The 75% identity threshold was selected empirically after running blastclust with a range of percent identity values: requiring more than 75% identity resulted in too many orthologues being split into multiple clusters, and allowing lower values resulted in too many clusters containing paralogues. When an orthologue in a draft assembly was split into two or more fragments, these were considered to be a single match.

ORFs identified only in the seven newly sequenced genomes by the JCVI automated sequence annotation pipeline were split at any run of five or more ambiguous amino acids ('X'), which resulted from in-frame strings of fifteen or more 'N' between contigs in the assemblies. After splitting, all ORFs and ORF fragments with length less than 50 aa were removed from the set. Amino acid sequences of the remaining ORFs were screened against the nucleotide sequences of the six previously available finished *Listeria *genomes using TBLASTN with an identity threshold of 65%, and those without any hits were identified. These novel *Listeria *ORFs were screened against all seven draft genome assemblies in order to determine their distribution across the set of strains.

### Core and pan genome analysis

The mixture model method of Snipen et al. [34] was used to estimate the number of genes in (i) the core genome (i.e., the set of genes found within every strain within the genus *Listeria *[34,35]) and in (ii) the pan-genome (i.e., the core genome plus the dispensable or accessory genome, which is defined as the genes that are found in some, but not all, strains including genes that are unique to a single strain [34,35]). To compare these estimates to another group of Gram-positive bacteria that includes closely related pathogens and non-pathogens, the same method was used to estimate the core and pan-genome of the *Bacillus cereus *group, a group that can be considered a single species from taxonomic point of view [36]. Only the chromosome sequences of five *B. anthracis*, nine *B. cereus*, two *B. thuringiensis*, and one *B. weihenstephanensis *strain were used for this analysis.

Cumulative pan-genome size plots for *Listeria *were calculated by selecting strains without replacement in random order 500 times, and then calculating the mean pan-genome size at each sampling point. Blast2GO [37] was used to perform a functional annotation of the genes found in the core and the pan-genome.

### Phylogenetic analysis

Phylogenetic trees of the *Listeria *isolates were constructed using sequences for 100 genes in the core genome of the species studied; these genes were randomly selected form all genes that were previously shown to have no evidence for either positive selection or recombination [38] as these two processes make it problematic to infer the true organismal phylogeny. Phylogenetic trees were inferred using neighbor joining, maximum parsimony and minimum evolution phylogenetic reconstruction methods available in the MEGA package version 4.1 [39]. A Bayesian analysis was performed using MrBayes 3.12 [40] and the GTR +I+G model of nucleotide evolution. A phylogenetic reconstruction using presence or absence data of genes in the pan genome was performed using the maximum parsimony method in PAUP* version 4.010b [41].

To infer the time to the most recent common ancestor of *Listeria *and to infer the approximate age of the individual *Listeria *species, a Bayesian molecular clock analysis was performed in BEAST version 1.5.2 [42] based on the concatenated 100 core genes. One strain from each species or lineage was included in the analysis. The molecular clock analysis was performed using a GTR +I+G nucleotide substitution model, and a relaxed clock model to account for variation in substitution rates. Tracer version 1.4.1 was used to assess the proper burn-in and sampling of the model parameters. We used a mutation rate of 4.5 × 10^-9 ^per site per year, as suggested by Ochman et al. [43] to calibrate the tree.

### Evolutionary analysis of internalins

As at least some internalins have been shown to be important virulence factors in *Listeria *(see the Background section for more details) we decided to employ a phylogenetic approach to infer the homology and evolution of genes in the internalin gene family. This was specifically important because the JCVI automated annotation pipeline annotated most internalin genes inconsistently as either (i) internalin A, (ii) leucine-rich repeat containing protein, or (iii) cell wall anchor domain-containing protein. Amino acid sequences of ORFs predicted to encode internalins or leucine-rich repeat proteins were extracted from the annotated genomes; the newly sequenced genomes were aligned to publicly available, well-annotated genomes (e.g., EGD-e and F2365) to identify internalin genes that were missed in the initial annotation. After alignment using the EINSI strategy in the MAFFT alignment package [44], internalin aa sequences were used for phylogenetic reconstruction using a maximum parsimony heuristic search in PAUP* 4.010b [41]. Gaps identified in internalin genes were either closed by PCR and Sanger sequencing or by reassembly of the raw SOLiD™ system reads using improved versions of the *de novo *assembly tools. Internalins found in *L. monocytogenes *CLIP80459 were not included in this analysis because the majority of the internalins (26 out of 28) in this strain are identical (at the aa level) to those found in *L. monocytogenes *F2365.

### Presence of virulence-associated genes

The presence, in the 13 genome sequences analyzed, of 78 putative virulence-associated genes previously reported by Camejo et al. [45] was assessed using whole genome alignments produced with Mauve 2.3.0. The PVclust R package [46] was used to perform a cluster analysis based on the presence/absence data for genes that showed variable presence/absence.

### Horizontal gene transfer and mobile elements

SIGI-HMM [47] was used to infer if genes in the genomes were acquired through horizontal gene transfer (HGT); this program uses a codon usage based hidden Markov model to infer if genes are of alien origin (i.e., have been introduced into the genome from a divergent gene pool by HGT). Genes with more than 10% ambiguous sites were excluded from the analyses. The detection sensitivity of SIGI-HMM was set to 0.95. Prophages and prophage derived regions were identified using the online version of Prophinder [48]. The Prophinder algorithm searches for regions that are dense in phage-like proteins, using a BLASTP search [33] against all phage proteins in the ACLAME database [49]. Transposons and plasmid related genes were identified with SIGI-HMM and through examination of the initial genome annotations for transposon- or plasmid-related genes.

### Identification of Restriction and Modification and CRISPR systems

Bacterial defense systems against mobile elements/foreign DNA implicated in the reduction of horizontal gene transfer include (i) restriction-modification (R-M) systems [50] and (ii) clustered, regularly interspaced, short palindromic repeat (CRISPR) systems [51]. R-M systems were identified by screening the initial annotation for genes involved in R-M systems. CRISPRfinder [52] was used to find CRISPR regions. Because the CRISPR regions consist of highly repetitive regions, which may prove problematic for short read based *de novo *assembly methods, the annotated genomes were also searched for CRISPR associated genes (Cas genes). When the presence of the actual CRISPR region was ambiguous, the presence of Cas genes was considered evidence for a functional CRISPR system.

### Caco-2 invasion assays

The ability to invade human intestinal epithelial cells, a phenotype associated with the presence of *inlA*, was tested for selected strains; the *L. marthii *strain was not tested as its invasiveness has previously been reported [1]. The invasion assays with the human intestinal epithelial cell line Caco-2 were performed as previously described [53]. *L. monocytogenes *10403S and isogenic Δ*inlA *(FSL K4-006) strain were included as controls in each invasion assay.

### Confirmation absence virulence associated genes in non-pathogenic strains

To confirm the absence of critical virulence associated genes (i.e. *inlA*, *inlB*, *inlC *and the *prfA *cluster) in genomes where these genes were not found, we resequenced the regions where these genes are found in pathogenic strains. The absence of *inlC *in these non-pathogenic strains was further confirmed by PCR with degenerate primers designed to amplify *inlC *in *L. monocytogenes *and *L. ivanovii*. Primer sequences and additional information can be found in additional file 1.

## Results

### De novo assembly of short sequence reads yields high quality draft genomes for selected *Listeria *species

**Figure 1:**
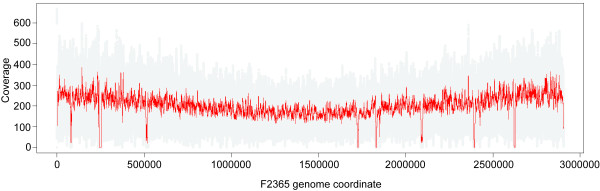
**Coverage of F2365 genome by R2-574 SOLiD™ system reads**. Depth of coverage of uniquely placed reads was plotted along the length of the *L. monocytogenes *F2365 chromosome. Gray dots indicate coverage at each base and the red line indicates the moving average with a window size of 1000. Uncovered gaps represent non-unique sequences, including the six rRNA operons.

The genomes of eight *Listeria *strains were sequenced, using mate-paired libraries, on the SOLiD™ 3 System. The previously sequenced *L. monocytogenes *strain F2365 (our strain ID FSL R2-574) was included in this set to evaluate assembly of SOLiD™ system reads. Mapping of FSL R2-574 SOLiD™ system reads to the F2365 reference genome resulted in 200× median unique coverage depth (Figure 1), with unique coverage gaps only in multicopy loci (e.g., rRNA genes). The corona_lite SNP calling tool identified 21 putative SNPs and 4 ambiguities (see additional file 2 for more information); 19 of these putative SNPs appeared to be legitimate based on coverage depth. PCR amplification and Sanger sequencing confirmed that all 21 putative SNPs represent real differences between the published genome sequence of F2365 and our isolate of this strain (FSL R2-574). The four ambiguities were found to be due to single base pair insertions or deletions in FSL R2-574 relative to the F2365 genome. Three additional mutational differences (two adjacent SNPs and a single base pair insertion) were found in FSL R2-574 during Sanger sequencing-based confirmation of the SNPs identified initially. Examination of the original trace files for the F2365 genome indicated that 24 of these 28 overall differences likely represent sequence errors in the original F2365 sequence (additional file 2). However, four SNPs (see additional file 2) appear to represent real differences between the F2365 and FSL R2-574 genomes, which most likely arose during laboratory passage, as suggested previously in both *L. monocytogenes *[54] and *Bacillus anthracis *[55].

Most of the FSL R2-574 genome was encompassed in eight large scaffolds with the largest scaffold over 1.4 Mb. Alignment of this assembly to the F2365 reference genome indicated that 98.09% of the genome was covered with identity of 99.64%, and fewer than ten misassemblies (i.e., juxtaposition in scaffolds of non-contiguous regions of the genome) were observed, indicating a high quality draft genome for FSL R2-574, according to the definition of Chain et al. [56].

Assembly of the SOLiD™ system reads resulted in 785 to 2,551 contigs per genome (Table 2); the sum of the contig lengths ranged from 2.8 to 3.2 Mb, which is comparable to genome sizes of previously sequenced *Listeria *genomes (Table 1). When non-contiguous genomic regions were found to be juxtaposed in the assembly, the scaffold was broken and reordered to correspond with the reference genome order. The number of potential misassemblies due to illegitimate scaffolding of contigs ranged from 68 (*L. seeligeri *FSL S4-171) to 500 (*L. monocytogenes *FSL F2-208) with a median of 152. Predicted ORF counts (based on JCVI annotations and orthology analyses) ranged from 2,724 for *L. marthii *FSL S4-120 to 3,017 for *L. seeligeri *FSL N1-067 (Table 2 and additional file 3).

**Table 2:** De novo assembly statistics for the 8 Listeria strains sequenced here

Strain	number of scaffolds	N50 scaffolds	number of contigs	N50 contigs	Estimated number of ORFs
***L. monocytogenes***					
FSL F2-208	1,437	49,992	2,531	2,639	2,910
FSL R2-574	163	1,433,496	1,538	3,241	ND^**a**^
***L. marthii***					
FSL S4-120	404	257,992	925	7,850	2,724
***L. innocua***					
FSL S4-378	896	102,515	1,837	4,230	2,885
FSL J1-023	324	247,625	790	9,133	2,737
***L. ivanovii***					
FSL F6-596	601	95,455	1,463	5,168	2,919
***L. seeligeri***					
FSL N1-067	343	282,765	785	10,831	3,017
FSL S4-171	216	226,677	868	5,655	2,820

^a ^ND = not done. This strain is a duplicate of *L. monocytogenes *F2365 and was included to assess the assembly methods used in this study.

### The *Listeria *pan-genome is estimated to contain approximately 6,500 genes, including about 2,000 core genes

**Figure 2:**
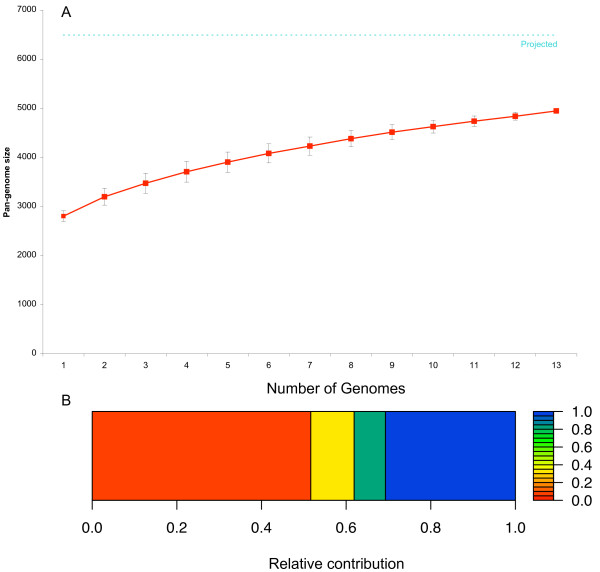
**Cumulative size and composition of the *Listeria *pan-genome**. (a) Cumulative pan-genome size plots were calculated by selecting strains without replacement in random order 500 times, and then calculating the mean pan-genome size at each sampling point (solid red line). Error bars indicate one standard deviation from the mean. Estimated pan-genome size from mixture model analysis is indicated as a dotted cyan line. (b) The graphical display of the mixture model represents the four components of the pan-genome as rectangles, including (i) a component of 31% of the genes with a detection probability of 1.0 (blue: the core-genome), (ii) a component representing 7% of the genes with a detection probability of 0.82 (teal), (iii) a component of 10% of the genes with a detection probability of 0.33 (yellow), and (iv) a component of 52% of the genes with a detection probability of 0.06 (rare genes: orange).

A total of 4,950 orthologous genes were found among the 13 genome sequences analyzed here, including the 7 new genome sequences generated. Based on these data, a mixture model approach [34] estimated the size of the actual *Listeria *pan-genome as 6,494 genes, suggesting that over 1,500 *Listeria *genes remain to be discovered by further sequencing (Figure 2a). According to this mixture model approach, the *Listeria *pan-genome best fitted a model with four components including (i) a component of 31% of the genes with a detection probability of 1.0 (the core-genome), (ii) a component of 7% of the genes with a detection probability of 0.82, (iii) a component of 10% of the genes with a detection probability of 0.33, and (iv) a component of 52% of the genes with a detection probability of 0.06 (Figure 2b). The lower Bayesian information criterion (BIC) of the four-component model (17,783) versus that of the three-component model (18,393) indicates a better fit. A relatively large part of the estimated pan-genome (76.2%) was covered by the actual pan-genome inferred from the 13 *Listeria *genome sequences. In contrast, only 42% of the estimated pan-genome was covered by the actual pan-genome inferred from the 17 genome-sequences of the *B. cereus *group.

**Figure 3:**
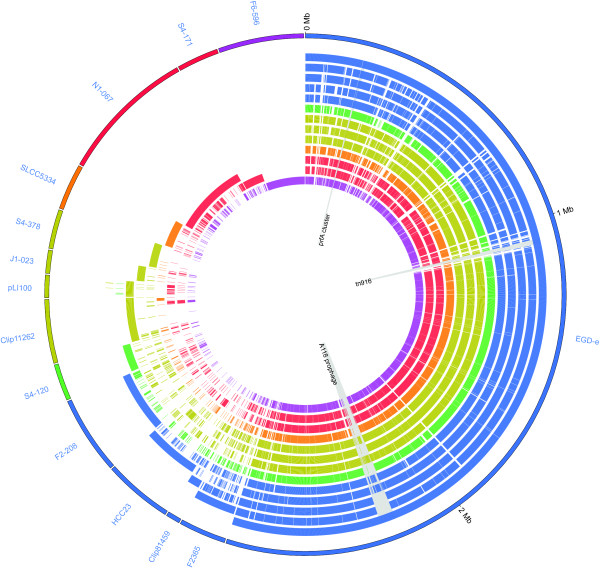
**Comparative genome content of 13 *Listeria *chromosomes and *L. innocua *plasmid pLI100**. The outermost circle indicates the source of each gene in the pan-genome with each gene represented by a constant width wedge. Starting at the top of the figure (0 Mb) and moving clockwise, all EGD-e genes are arranged in chromosomal order. Continuing clockwise, all genes not present in EGD-e are grouped by strain (as indicated by segment labels). Genes in the F2365 segment are present in F2365, but absent from EGD-e, and genes in the Clip81459 segment are present in Clip81459, but absent from F2365 and EGD-e, and so on. In this way, each gene is represented only once in the diagram. Gene order in all segments except EGD-e is monotonically increasing, but discontinuous, since shared genes may be represented in other segments. Internal circles indicate gene presence (solid color) or absence (unfilled) of each gene in each of the 13 strains examined. Circles from outer to inner are in the same order as strains on the outer circle, starting with EGD-e, followed by F2365, etc. *L. monocytogenes *strains are in blue; *L. marthii *is in green; *L. innocua *strains are in gold; *L. welshimeri *is in orange; *L. seeligeri *strains are in red; *L. ivanovii *subsp. *londoniensis *is in purple. The location, in the EGD-e genome, of the *prfA *virulence cluster, conjugative transposon tn916 and prophage A118 are specifically indicated. This figure was created using the Circos software [85].

The observed core genome shared by all 13 *Listeria *strains comprises 2,032 genes (shown as genes found in EGD-e and all other genomes in Figure 3), while the estimated size of the core genome is 1,994 genes, indicating that the core genome as defined by this study will change very little as more genomes are sequenced.

While the number of accessory genes observed so far is 2,918, the estimated total number of accessory genes for the genus *Listeria *(except *L. grayi*) is 4,500. Only a small proportion of ORFs in the different *Listeria *genomes was predicted to be introduced by HGT (2.0 to 6.4%; see Table 3) and only one of these ORFs, a collagen binding protein in *L. monocytogenes *FSL F2-208 is potentially associated with virulence. The majority of the genes found in the core genome are involved in metabolic processes (nucleobase, nucleoside, nucleotide and nucleic acid metabolic processes [17% of GO hits], cellular macromolecule metabolic processes (14% of GO hits) and protein metabolic processes [10% of GO hits]) and transport (13% of GO hits), which is congruent with the general notion that the core genome contains genes that are essential for the survival of the organism. Genes involved in metabolic processes and transport also dominate the accessory genome (nucleobase, nucleoside, nucleotide and nucleic acid metabolic processes [21% of GO hits], cellular macromolecule metabolic processes (20% of GO hits) and transport [13% of GO hits]), which can be explained by the fact that species in *Listeria *have a primarily saprotrophic lifestyle and genes in the accessory genome are putatively involved in the metabolism of specific carbon sources. A large part of the accessory genome (35% of the genes), however, cannot be classified according to the Gene Ontology or is without any significant Blast hits to proteins currently in Genbank. Among these unclassified genes in the accessory genome are hypothetical proteins, proteins involved in phage resistance and prophage associated genes.

**Table 3:** Overview of selected genome characteristics of Listeria genomes used for comparative analysis

Strain	**No. of strain specific ORFs**^**a**^	No. of internalin genes	**% ORFs introduced by HGT**^**b**^	**No. of prophages**^**c**^	**No. of monocins**^**d**^	**R-M system**^**e**^	**CRISPR**^**f **^**presence**	Plasmid presence	**No. of Transposons**^**g**^
***L. monocytogenes***									
F2365	8	26	3.4	0	1	II	no	no	0
EGD-e	36	25	3.4	1*	1	-	yes	no	1
CLIP81459	9	28	3.2	0	1	I	no	no	0
HCC23	44	18	4.8	3	0	II	yes	no	0
FSL F2-208	101	23	4.6	1*	1	I	yes	no	1
***L. marthii***									
FSL S4-120	74	19	6.4	0	1	I	yes	no	0
***L. innocua***									
CLIP11262	159	20	4.0	5*	1	I	yes	yes	0
FSL S4-378	108	19	5.9	2	1	I	yes	no	0
FSL J1-023	47	17	4.5	0	1	II (2)	no	no	0
***L. welshimeri***									
SLCC5334	113	9	3.6	1	0	I	no	no	1
***L. ivanovii***									
FSL F6-596	230	20	2.0	1	2	-	yes	no	0
***L. seeligeri***									
FSL S4-171	91	17	3.0	1*	2	I	no	no	0
FSL N1-067	234	15	4.3	2*	1	I & III	yes	yes	0

^a ^ORFs were designated as 'strain specific' if they were only present in one strain (based on comparisons with the other genomes included in our study here

^b ^These ORFs were detected as derived from horizontal gene transfer by the SIGI-HMM program [47].

^c ^An asterisk indicates that one of the prophages is inserted in *comK*.

^d ^The number of regions encoding monocins, defective prophage or satellite prophages.

^e ^I = Type I Restriction-Modification (R-M) system, II = Type II R-M system, III = Type III R-M system, - = no R-M system.

^f ^CRISPR stands for Clustered, regularly interspaced, short palindromic repeat loci.

^g ^The integrative and conjugative element found in the genome of *L. welshimeri *(Genbank accession AM 263198: lwe0767-lwe0796) was not reported in the original publication of this genome.

### *L. seeligeri *genome characteristics

**Figure 4:**
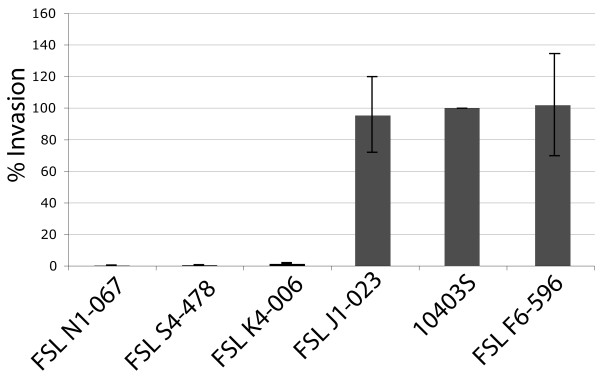
**Invasion efficiencies in Caco-2 cells of *Listeria *strains**. The strains tested are shown on the x-axis and include *L. seeligeri *FSL N1-067, *L. innocua *FSL S4-378 (non-hemolytic), *L. monocytogenes *10403S Δ*inlA *(FSL K4-006), *L. innocua *FSL J1-023 (hemolytic) and *L. ivanovii *subsp. *londoniensis *FSL F6-596 (ATCC 49954). Invasion efficiency (the number of recovered cells/number of cells used for inoculation) was normalized to the invasion efficiency obtained for *L. monocytogenes *10403S, which was set as 100%, and was included as a control strain in each essay. Three independent invasion assays were performed for each strain tested.

A total of 3,017 and 2,820 ORFs were identified in *L. seeligeri *FSL N1-067 and FSL S4-171, respectively (Table 2); these ORF counts are considerably higher than the 2,710 ORFs recently reported for *L. seeligeri *SLCC3954 [14]. In addition to 88 *L. seeligeri *specific ORFs (i.e., ORFs only found in both *L. seeligeri *FSL N1-067 and FSL S4-171), which included three ORFs encoding specific internalin-like genes, we also identified strain specific ORFs (Table 3), including seven and three genes that encode putative internalins (in FSL S4-171 and FSL N1-067, respectively). Overall, 15 and 17 internalin genes were found in *L. seeligeri *FSL N1-067 and FSL S4-171, respectively (Table 3); by comparison 16 internalin-like genes were reported for *L. seeligeri *SLCC3954 [14]. The genomic region harboring *inlAB *in other *Listeria *is completely absent from the *L. seeligeri *genomes (see additional file 4). The *inlGHE *region is absent from the FSL N1-067 genome; in FSL S4-171 this region contains ABC-transporter encoding ORFs (see additional file 5). Consistent with the absence of *inlA*, *L. seeligeri *FSL N1-067 was non-invasive in the Caco-2 cell invasion assay, with invasion efficiencies similar to the *L. monocytogenes *10403S *inlA *null mutant (Figure 4).

The *L. seeligeri *FSL N1-067 *prfA *cluster (additional file 6) is very similar to the *prfA *cluster previously described [3] for *L. seeligeri *with the exception that instead of a duplication of *plcB*, two short open reading frames (encoding two proteins of 51 and 61 aa), with 62% and 41% similarity to parts of a *L. seeligeri plcB *sequence are found (GenBank accession AAR97365). The non-hemolytic strain FS S4-171 is characterized by the absence of the *prfA *cluster as also confirmed by PCR (additional file 6); however, the two ORFs (ORFX and ORFI) reported to be lost in the non-hemolytic isolates analyzed by Volokhov et al. [24] are present in the remains of the FSL S4-171 *prfA *cluster (additional file 6).

Evidence for the presence of a plasmid was found for the hemolytic *L. seeligeri *FSL N1-067; the initial annotation identified six ORFs that were annotated as two resolvases, a replication associated protein and a LtrC-like conjugative protein. BLAST searches of these plasmid-related and adjacent proteins revealed a high identity to proteins encoded on the *L. innocua *CLIP11262 plasmid pLI100. The sequence of this plasmid was used as a query to identify more plasmid-derived contigs in *L. seeligeri *FSL N1-067. P-RAST annotation [57] of these contigs and comparison to the pLI100 plasmid identified plasmid related contigs that encoded 63 putative ORFs, including 44 ORFs with homology to genes found in pLI100 (28 of these ORFs with aa identity to pLI100 ORFs of ≥ 95%). These contigs totaled 60,916 bp, representing an estimate of the minimum plasmid size (use of pLI100 as a query may not have identified non-homologous plasmid regions). Some of the predicted genes on this plasmid are involved in cadmium, arsenic and multi-drug resistance, consistent with previous reports of plasmid-associated cadmium and arsenic resistance genes in *L. monocytogenes *[58]. Two different R-M systems were found in the genome of *L. seeligeri *FSL N1-067; a MjaXIP specific Type I system and an EcoPI specific Type III system. Only one Type I restriction modification system was found in the non-hemolytic *L. seeligeri *strain FSL S4-171. A CRISPR system was identified in FSL N1-067, but not in FSL S4-171 (Table 3), suggesting differences in phage resistance between the two strains.

### *L. ivanovii *subsp. *londoniensis *genome characteristics

A total of 2,919 ORFs were identified in the *L. ivanovii *subsp. *londoniensis *genome, which contains one prophage and two monocin-like regions (Table 3). Presence of a functional CRISPR system was inferred from the presence of Cas genes. The *L. ivanovii *genome included 20 genes that putatively encode internalins (Table 3); the *inlAB *region (additional file 4) contains seven ORFs, including five that encode internalins (i.e., one *inlA *and two *inlB *homologues, one internalin A-like protein and an internalin with distant homology to lin2724, an internalin found in *L. innocua *CLIP11262). Consistent with the presence of an *inlA *homologue, *L. ivanovii *subsp. *londoniensis *FSL F6-596 showed Caco-2 cell invasion efficiency comparable to *L. monocytogenes *10403S (Figure 4).

### *L. innocua *genome characteristics

A total of 2,855 and 2,737 ORFs were identified in the genome sequences for the non-hemolytic *L. innocua *FSL S4-278 and the hemolytic *L. innocua *FSL J1-023. Only three genes were exclusively found in all three *L. innocua *genomes (i.e., the two genomes sequenced here and CLIP11262); lin0464, which encodes a putative transcriptional regulator and lin1452 and lin2741, which both encode hypothetical proteins. Noteworthy among the strain specific ORFs (Table 3) are three ORFs encoding genes involved in cobalt transport systems in strain FSL J1-023. No prophage regions were identified in the genome of the hemolytic *L. innocua *strain FSL J1-023, while the non-hemolytic strains CLIP11262 and FSL S4-378 contain five and four prophage regions, respectively. The number of internalin genes in the *L. innocua *genomes ranged from 17 to 20 (Table 3). The *inlAB *region of the hemolytic *L. innocua *only contains a homologue of *inlA*; *inlB *is absent as previously reported [23]. Consistent with these findings, the hemolytic *L. innocua *FSL J1-023, which contains *inlA*, shows average Caco-2 invasion efficiencies comparable to *L. monocytogenes *10403S (Figure 4), while the non-hemolytic *L. innocua *FSL S4-378 was non-invasive, with invasion efficiencies similar to those for the *L. monocytogenes *10403S *inlA *null mutant (Figure 4).

Modification and restriction systems were present in all three *L. innocua *genomes (Table 3). *L. innocua *CLIP11262 and FSL S4-378 harbor a type I R-M system, while the hemolytic *L. innocua *FSL J1-023 has two type II R-M systems, a Sau3AI specific system and an EcoRV specific system (which is unique to this strain). CRISPR systems are present in the genomes of CLIP11262 and FSL S4-378, but were not found in FSL J1-023.

### *L. marthii *genome characteristics

A total of 2,724 ORFs were identified in the *L. marthii *FSL S4-120 genome, including 74 ORFs exclusively found in this strain (Table 3). Prophinder found no evidence for the presence of prophages, however one monocin region was detected. Among the genomes examined here, the *L. marthii *genome has the highest percentage (6.4%) of ORFs that are introduced through HGT (Table 3). One of the regions introduced by HGT in *L. marthii *is a genomic island that encodes for part of a lantibiotic biosynthesis gene cluster. We also identified 23 genes that were only found in the genomes of *L. marthii *and the *L. monocytogenes *lineage IIIC strain FSL F2-208; BLAST searches against GenBank revealed that the eight of these genes were also found in other *L. monocytogenes *lineage III and IV genomes (2 and 6 genes in FSL J2-072 and FSL J1-208, respectively). While 19 internalin-like genes were found in the *L. marthii *genome, the *inlAB *region did not contain any internalin-like genes as confirmed by PCR and the *inlGHE *region contained one ORF encoding an ABC transporter and a homolog of *inlC2 *(additional file 5). The *L. marthii *genome contains a type I R-M system and a CRISPR system.

### *L. monocytogenes *genome characteristics

A total of 2,910 ORFs were identified in *L. monocytogenes *lineage IIIC strain FSL F2-208 (Table 3), which is similar to the number of ORFs identified in other *L. monocytogenes *genomes. Only four genes were shared between all *L. monocytogenes *genomes but not found in the genomes of any of the other species; LMOf2365_0100 (a MerR transcriptional regulator), LMOf2365_0101 (an aldo/keto reductase family oxidoreductase), LMOf2365_0477 (a hypothetical protein) and LMOf2365_0769 (a DNA binding protein). While the number of prophages identified ranged from zero to three (Table 3), all *L. monocytogenes *genomes contained one monocin region, except HCC23, which seems to lack a monocin region. The chromosome of *L. monocytogenes *FSL F2-208 contains a region with high similarity to a putative conjugative element CTn1 found in *Clostridium difficile *[59]; this region contains one ORF encoding a putative virulence factor, a collagen adhesion protein. The number of internalin-like genes in the *L. monocytogenes *genomes ranged from 18 to 28 (Table 3). The *inlAB *region is highly variable, however all genomes examined here contain ORFs with homology to *inlA *and *inlB *(additional file 4). The *inlGHE *region is completely absent from the HCC23 genome; in FSL F2-208, this region seems to only contain an *inlC2 *homolog (additional file 5). All *L. monocytogenes *genomes, with the exception of the EGD-e genome, contain R-M systems (see Table 3).

### Phylogenetic analyses identify sister groups containing pathogenic and non-pathogenic *Listeria *species

**Figure 5:**
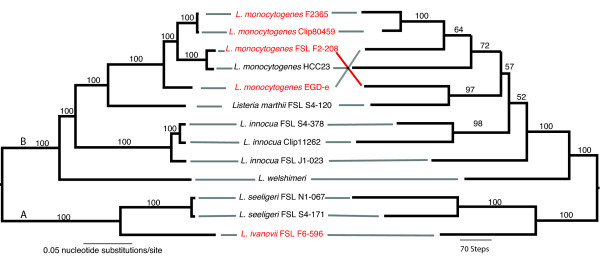
**Comparison of phylogenetic trees based on *Listeria *core gene sequences and genomic gene content**. The 100 gene sequence tree (left) was inferred using Bayesian phylogenetic inference (MrBayes v. 3.12) and the values above the branches are the posterior probabilities. The gene content tree based on the presence/absence of 4950 orthologous genes (right) was inferred using maximum parsimony and the values above the branches are bootstrap values based on 1000 bootstrap replicates. Pathogenic strains are colored red, while non-pathogenic strains are black.

Alignment of the 100 concatenated genes that were used for phylogenetic reconstruction comprised 90,215 nucleotide sites, including 27,945 variable sites; the average pairwise nucleotide identity based on the 100 genes was 84.8%. All methods except maximum parsimony yielded similar topologies, placing *L. seeligeri *and *L. ivanovii *in one well-supported clade (100% bootstrap, 100% posterior probability) and *L. welshimeri *basal to another well supported clade containing *L. innocua*, *L. marthii *and *L. monocytogenes *(see Figure 5, the left tree shows the phylogeny resulting from the Bayesian analysis). Within the *L. innocua*/*L. marthii*/*L. monocytogenes *clade, *L. innocua *is the most divergent species, while *L. marthii *forms a sister group to *L. monocytogenes*. All phylogenetic relationships within this clade are well supported (bootstrap support > 98%, 100% posterior probability). The maximum parsimony tree differs from the other trees by its placement of *L. welshimeri *within the *L. seeligeri*/*L. ivanovii *clade. While neighbor joining, maximum likelihood, minimum evolution and parsimony methods do not find any significant bootstrap support for the placement of *L. welshimeri *in the phylogeny, the Bayesian analysis supports placement of *L. welshimeri *as a basal taxon to the *L. innocua*/*L. marthii*/*L. monocytogenes *clade with a highly significant posterior probability (100%).

A maximum parsimony phylogeny based on the presence and absence of orthologous genes yielded in a single tree (4,805 steps, consistency index 0.608, rescaled consistency index 0.446; Figure 5, right tree) with topology similar to the tree based on Bayesian, minimum evolution, maximum likelihood and neighbor joining phylogenetic inference of the 100 gene sequence dataset. The only differences are the placement of *L. monocytogenes *FSL F2-208 and *L. innocua *FSL J1-023. In the gene content-based phylogeny, the hemolytic *L. innocua *FSL J1-023 strain is placed is outside of the clade containing the two non-hemolytic *L. innocua *strains. *L. monocytogenes *FSL F2-208 is placed in a well-supported (97% bootstrap support) clade together with *L. marthii*. This placement can be mainly attributed to 23 genes that were only found in the *L. monocytogenes *FSL F2-208 genome and the *L. marthii *genome (see section '*L. marthii *genome characteristics' for more information).

**Figure 6:**
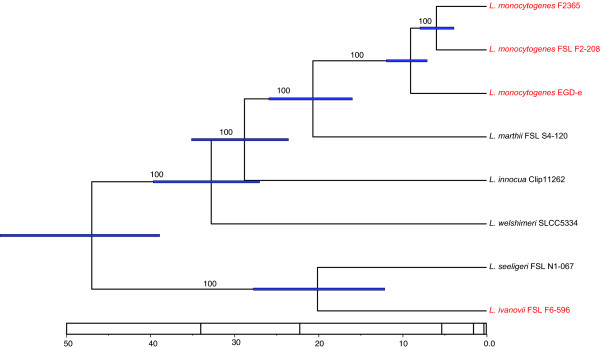
**Maximum clade credibility tree summarizing the results of the Bayesian molecular clock analysis of the 100 concatenated core genome genes**. The timeline indicates the age of the nodes when a mutation rate of 4.5 × 10^-9 ^per year/site was used to calibrate the tree. One strain from each species or lineage was included in the analysis; *L. monocytogenes *lineages I (F2365), II (EGD-e) and IIIC (FSL F2-208), *L. marthii *(FSL S4-120), *L. innocua *(CLIP11262), *L. welshimeri *(SLCC5334), *L. seeligeri *(FSL N1-067), and *L. ivanovii *subsp. *londoniensis *(FSL F6-596). Values above the branches indicate posterior probability values, blue horizontal bars on the nodes show the 95% highest probability density of the inferred age of the nodes. The posterior probability of the individual trees and 95% highest probability density of the divergence time were based on 9,000,000 post burn-in generations of a 10,000,000 generation run. The values on the time lines represent ages as million years before present. Labels of pathogenic strains have been colored red.

A molecular clock analysis of the 100-gene phylogeny, calibrated with the mutation rate proposed by Ochman et al. (Figure 6), places the time of most recent common ancestor (MRCA) of *Listeria *(excluding *L. grayi*) at 47 million years ago (mya) with a 95% highest probability density (HPD) of 58 mya to 39 mya. The time of divergence of *L. welshimeri *from the MRCA of *L. innocua*, *L. marthii *and *L. monocytogenes *was estimated at 33 mya (95% HPD: 40-27 mya). *L. innocua *is estimated to have diverged from the MRCA of *L. monocytogenes *and *L. marthii *at 29 mya (95% HPD: 35-24 mya). *L. marthii *and *L. monocytogenes *were estimated to have diverged from each other around the same time as the divergence of *L. seeligeri *and *L. ivanovii *(21 and 20 mya, respectively). Because the mutation rate proposed by Ochman et al. [43] is based on the divergence of *Escherichia coli *and *Salmonella enterica*, it may not be applicable to other bacteria. We therefore used the sequence divergence of 16S rRNA between *L. monocytogenes *and *L. ivanovii *to infer the putative age of the MRCA of *Listeria*. The divergence between *L. monocytogenes *and *L. ivanovii *is 0.9 to 1.2% (calculated based on full length 16S rDNA data previously reported [1]), which would translate to dating the MRCA at 45 to 60 mya, given the universal 16S rRNA divergence rate of 1% per 50 million years proposed by Ochmann and Wilson [60].

### While non-pathogenic *Listeria *have fewer virulence associated genes, gene presence patterns for these genes identify only internalin C as a gene that differentiates pathogenic and non-pathogenic *Listeria*

Out of 78 putative virulence-associated genes previously reported [45] (see additional file 7), 56 were found in all 13 genomes analyzed. Only 22 of these genes (not including the genes in the *prfA *cluster) were variable in their presence between genomes (Table 4), including three putative virulence-associated genes that were found solely in the *L. monocytogenes *EGD-e genome. Using a hierarchical cluster analysis based on the presence or absence of the 22 variable genes, the strains were subdivided into two clusters, including (i) a cluster composed of *L. ivanovii *subsp. *londoniensis*, all *L. monocytogenes *strains and the hemolytic *L. innocua *strain and (ii) a cluster composed of only non-pathogenic strains (the non-hemolytic *L. innocua *strains, *L. seeligeri*, *L. marthii *and *L. welshimeri*).

**Table 4:** Variable virulence associated genes other than those found in the prfA cluster and their distribution in the genomesa

Gene designation	EGD-e homologue	**Lm**^**b **^**EGD-e**	**Lm**^**b **^**F2365**	**Lm**^**b **^**CLIP 80459**	**Lm**^**b **^**lineage IIIA**	**Lm**^**b **^**lineage IIIC**	**Li**^**b **^**hly+**	**Liv**^**b**^	**Ls**^**b **^**hly+**	**Ls**^**b **^**hly-**	**Lma**^**b **^**hly-**	**Li**^**b **^**hly-1**	**Li**^**b **^**hly-2**	**Lw**^**b **^**hly-**
lmo0206	lmo0206	+	+	+	+	+	+	-	-	-	-	-	-	-
lmo0257	lmo0257	+	+	+	+	+	+	+	+	+	+	-	-	+
*inlH*/*inlC2*	lmo0263	+	+	+	-	+	-	-	-	-	-	-	-	-
*vip*	lmo0320	+	+	+	-	-	-	-	-	-	-	-	-	-
*inlA*^*a*^	lmo0433	+	+	+	+	+	+	+	-	-	-	-	-	-
*inlB*^*a*^	lmo0434	+	+	+	+	+	-	+	-	-	-	-	-	-
*uhpT*	lmo0838	+	+	+	+	+	+	+	+	+	-	-	-	-
lmo0915	lmo0915	+	+	+	+	+	+	+	-	+	+	+	+	+
*aut*	lmo1076	+	+	+	+	-	+	+	+	+	+	+	+	+
lmo1081	lmo1081	+	-	-	-	-	-	-	+	-	-	-	-	-
lmo1082	lmo1082	+	-	-	-	-	-	-	+	-	-	-	-	-
lmo1099	lmo1099	+	-	-	-	-	-	-	-	-	-	-	-	-
lmo1102	lmo1102	+	-	-	-	-	-	-	-	-	-	-	-	-
lmo1290	lmo1290	+	+	+	+	+	-	+	-	+	+	-	-	-
*inlC*^*a*^	lmo1786	+	+	+	-	+	-	+	-	-	-	-	-	-
lmo2026	lmo2026	+	-	-	-	-	-	-	-	-	-	-	-	-
*bsh*	lmo2067	+	+	+	+	+	-	-	-	-	-	-	-	-
lmo2157	lmo2157	+	+	+	-	-	-	+	-	-	+	-	-	-
lmo2439	lmo2439	+	+	+	+	+	+	+	+	-	+	+	+	+
*ami*	lmo2558	+	+	+	+	+	-	-	+	+	+	+	+	+
lmo2713	lmo2713	+	+	+	+	+	+	-	+	+	+	+	+	+
*inlJ*	lmo2821	+	+	+	-	+	-	-	-	-	-	-	-	-

^a ^The genes listed here are based on 78 putative virulence associated genes reported by Camejo et al. [45]. Only 22 genes found to show variable presence among the 13 genomes analyzed here are shown. The absence of the prfA-cluster, inlA, inlB and inlC was additionally confirmed by PCR and in some cases sequencing (see additional file 1 for more information).

^b ^Lm = *L. monocytogenes*; Li hly-1 = *L. innocua *CLIP 11262 (hemolysis negative); Li hly-2 = *L. innocua *FSL S4-378 (hemolysis negative); Li hly+ = *L. innocua *FSL J1-023 (hemolysis positive); Lma = *L. marthii *FSL S4-120; Lw = *L. welshimeri *SLCC5334; Liv = *L. ivanovii *subsp. *londoniensis *ATCC 49954; Ls hly+= *L. seeligeri *FSL N1-067 (hemolysis positive); Ls hly-= *L. seeligeri *FSL S4-171 (hemolysis negative).

Other than the *prfA *virulence cluster, the only gene that is absent from strains in the non-pathogen cluster but present in the first cluster is *inlA*, encoding internalin A, a key determinant of host intestinal cell invasion. The *inlC *gene, encoding internalin C, is the only gene that differentiates pathogens (i.e., *L. monocytogenes *EGD-e, *L. monocytogenes *F2365, *L. monocytogenes *FSL F2-208 and *L. ivanovii *subsp. *londoniensis*) from non-pathogens (all other strains). There is a significant (*p *< 0.05, Wilcoxon test) difference between pathogenic and non-pathogenic strains in the number of variable virulence-associated genes, with 10 to 22 genes present in pathogenic strains and 5 to 12 genes present in non-pathogenic strains.

### All *Listeria *strains contain multiple internalin-encoding genes with 16 internalin genes present in both main clades of the genus *Listeria*

A total of 252 putative internalin genes were identified in the genomes examined here; the number of internalin genes ranged from 9 internalins in *L. welshimeri *SLCC5334 to 28 internalins in *L. monocytogenes *CLIP81459 (Table 3). The number of internalin genes in the non-pathogenic strains (Table 1) was significantly lower as compared to the number in pathogenic strains (*p *< 0.05, Wilcoxon test). Within the internalin gene tree (additional file 8), three main clades can be recognized, including (i) a clade containing *inlA*, *inlB*, *inlC*, *inlF*, internalin genes found in the *inlGHE *region and related internalin genes, (ii) a clade containing *inlI *and *inlJ*, and (iii) a clade of genes encoding mainly uncharacterized internalin-like proteins. None of these three clades is supported by a significant posterior probability. We also identified 16 clades (marked in red in additional file 8), all with a significant posterior probability (>95%), that each contain genes from isolates of both the *L. monocytogenes*/*L. marthii*/*L. innocua *clade (clade A, Figure 5) as well as the *L. seeligeri*/*L. ivanovii *clade (clade B, Figure 5), supporting a MRCA that contained these internalin genes. Although some of these 16 internalin gene clades (e.g., the *inlA *clade) contained only a single sequence for one of the two *Listeria *clades, the internalin genes found in *L. ivanovii *and *L. seeligeri *(i.e., clade B) are generally highly divergent from their clade A homologues (see additional file 8), which supports presence of these genes in the MRCA as opposed to introduction by horizontal gene transfer.

## Discussion

### High quality draft genomes can be obtained through de novo assembly of short read sequences

The emergence and maturation of next generation sequencing (NGS) technology, driven in large part by efforts to develop approaches that allow for completion of a human genome for $1,000 [61], has made possible rapid, inexpensive, and high-throughput microbial whole-genome analysis, which promises to improve our understanding of bacterial pathogenesis, and our ability to detect and control infectious diseases. Our data show that NGS data facilitate *de novo *assembly and analyses of bacterial genomes. Although these draft genomes can have up to thousands of sequence gaps, the quality of the assembly is sufficient for automated annotation, and subsequent comparative genomics analyses, particularly when studying a conserved group of organisms such as the *Listeria *species examined here. Thus, NGS represents a feasible approach for rapid and comprehensive pathogen identification, subtyping, source tracking, and surveillance [62], and has the potential to be developed, in the long term, into routine diagnostic applications. The utility of draft genomes for identification of candidate vaccine targets has also been recently demonstrated [63].

### The genus *Listeria *sensu stricto has a pan-genome characterized by limited introduction of new genetic material with 2,032 core and 2,918 accessory genes identified to date

Our data show that the members of the genus *Listeria *have a highly conserved genome with limited acquisition, from other gene pools, of homologous and non-homologous genes, even though horizontal transfer of homologous genes within and between *Listeria *species has clearly been shown to occur [38,64]. Although the pangenome of the genus *Listeria *is not closed, there seems to be very limited on-going introduction of new genetic material from external gene pools (i.e., other genera). Data supporting this limited introduction of new genetic material into the pangenome include (i) the observation that the core and accessory genes identified among the 13 genomes analyzed represent a large proportion (i.e., 76.2%) of the predicted pan-genome, (ii) the similarity in size of observed core genome (2,032 genes) and predicted core genome (1,994 genes), suggesting limited gene loss and deletion, (iii) highly conserved estimated genome size (from 2.8 to 3.2 Mb), (iv) a relatively small fraction (4% on average) of genes that have atypical codon usage, and (v) a small number of prophages and transposons. The fact that most of these prophages have a codon usage pattern that is similar to their host indicates that they have co-evolved with their *Listeria *hosts [65]. A *Listeria *pan-genome characterized by limited introduction of genetic material is also supported by the observation that pan-genome coverage for the genus *Listeria *(except *L. grayii*) is higher than the pan-genome coverage estimates for most bacterial species, which range from 30% (for *Escherichia coli*, based on genomes of 22 strains) to 73% (for *Francisella tularensis*, based on genomes of 7 strains) [34]. A pan-genome coverage estimate performed for the *Bacillus cereus *group, a group of closely related pathogenic and non-pathogenic Gram-positive species, revealed a coverage of 42%, indicating pan-genome coverage of *Listeria*, is also high compared to Gram-positive organisms. The *Bacillus cereus *group, however, can be considered a single species from a taxonomic perspective [36]. In the case of *Listeria *this measure of shared gene content should not be interpreted to mean that *Listeria *species are very closely related and may in fact comprise one species. On the contrary, the *Listeria *species have diverged substantially in the primary sequence of their core genes with an average pair-wise nucleotide identity of 84.8%, compared to average pair-wise nucleotide identities within species of 99.2% in *F. tularensis *[66] and 96.7% in *E. coli *[67]. Phillipy et al. [68] predicted a closed pan-genome for the species *L. monocytogenes*, which is congruent with our observations for the complete genus.

The mechanism behind the limited occurrence of gene acquisition from outside gene pools in *Listeria *remains to be determined. Although several strains harbor an insertion of prophage A118 in the *comK *open reading frame, which encodes a transcriptional regulator of competence, *comK *is intact in *L. marthii*, *L. innocua *FSL J1-023 and FSL S4-378, and *L. ivanovii *subsp. *londoniensis*, as well as the previously sequenced *L. monocytogenes *F2365 and HCC23 genomes. While most of the competence related genes are present in all *Listeria *genomes [69] and while evidence for homologous recombination has been detected by multiple studies [38,64,70], natural competence has not yet been report for any *Listeria *strains [11]. Limited natural competence may thus at least partially explain the low level of gene acquisition from outside gene pools, particularly since our data suggest that most listeriophages do are part of the closed *Listeria *pangenome. In addition, limited gene acquisition in the genus *Listeria *may also be facilitated by the presence, in all genomes of the genus examined so far, of well-developed defense system against foreign DNA/mobile elements, including R-M systems [71] and/or CRISPR systems [51]. Both systems have been shown to limit or block horizontal gene transfer in *Staphylococcus aureus *[72,73]. This would explain why functional transposable elements are virtually absent from *Listeria*, and if present (as is the case for the conjugative elements reported here) contain a putative anti-restriction gene, which protects them from the restriction modification system.

Despite the overall high conservation of genome content across different *Listeria *species, gene loss and deletion events, as well as introduction of genetic material through horizontal gene transfer from other gene pools occurs in this genus, often with phenotypic consequences. For example, the chromosomal region that contains *inlAB *in *L. monocytogenes *and *L. ivanovii *appears to be hypervariable with evidence for deletion events (e.g., in *L. seeligeri*) and horizontal introduction of genetic material from other genera (e.g., the presence, in the *L. ivanovii inlAB *region, of two ORFs with relatively high similarity to *Enterococcus *genes and the presence, in the *inlAB *region of *L. marthii*, of approximately 15 ORFs that were putatively introduced by horizontal gene transfer), consistent with another report [13] that also suggested putative horizontal gene transfer events in this region.

### While *Listeria *includes a number of species-like clades, many of these putative species include subclades or strains with atypical virulence-associated characteristics and gene profiles

Generally, within the genus *Listeria*, only members of the species *L. monocytogenes *are considered to be human pathogens, while members of the species *L. ivanovii *are considered to be animal pathogens [7]. Key genes that clearly contribute to virulence, as supported by experimental evidence, include (i) genes located in the *prfA *cluster, which are critical for intracellular survival and cell to cell spread [17], (ii) *inlA *and *inlB*, which are critical for invasion of intestinal epithelial and hepatic cells, respectively [74], and (iii) *inlC*, which encodes a protein that is specifically required for cell-to-cell spread [21]. Strains representing *L. monocytogenes *lineages I, II, and III as well as the *L. ivanovii *subsp. *londoniensis *strains contained the full complement of these virulence genes (i.e., *prfA *cluster, *inlAB*, *inlC*), consistent with the experimentally verified virulence of these organisms [75,76]. Our full genome analyses suggest that the evolution from a *Listeria *ancestor that contained all three virulence loci yielded species and strains that have lost one or more of these key virulence genes. *L. welshimeri*, the majority of *L. innocua *strains, and non-hemolytic *L. seeligeri *strains lack all three of these virulence loci, consistent with their observed avirulence [22,77].

Other strains are lacking only a subset of the key virulence genes found in most *L. monocytogenes *and *L. ivanovii*. Hemolytic *L. seeligeri *carries the *prfA *cluster, but lacks *inlAB *and *inlC*, consistent with its avirulence in mammalian tissue culture and animal models [77]. Interestingly, some strains (represented by the hemolytic *L. innocua *strain characterized here) contain the *prfA *cluster as well as *inlA *and have the ability to invade human intestinal epithelial cells, while lacking *inlC *and showing avirulence in a mouse model [22]. Similarly, at least one *L. monocytogenes *strain (HCC23, representing lineage IIIA) contains the *prfA *cluster as well as *inlAB*, while lacking *inlC *and showing avirulence in mouse infection experiments [78]. These strains represent a particular challenge for virulence classification, as they would typically be classified as virulent with standard assays (as they are hemolytic and positive for some key virulence genes). Overall, our data, along with previously reported virulence characterizations of isolates representing different *Listeria *species as well as atypical strains (e.g., hemolytic *L. innocua*) [22-24], clearly indicate the need for a well-designed molecular approach to define pathogenic strains within the genus *Listeria*. While we hypothesize that use of multiple marker genes, e.g., genes in the *prfA *cluster, *inlA *(including identification of virulence attenuating premature stop codons [53]), *inlB*, and *inlC *is needed to identify virulent strains, further tissue culture and animal studies are needed to confirm appropriate marker genes. In addition, further comparative genomics studies of phenotypically variable *Listeria *will be needed to identify and validate diagnostic targets and markers.

### The genus *Listeria *represents two main clades that diverged from a common ancestor that contained the *prfA *cluster and a number of internalin genes, most likely 47 million years ago

The use of 100 core genes that have been previously shown to show no evidence for positive selection nor homologous recombination resulted in a robust phylogeny dividing *Listeria *(except *L. grayi*) into two main clades; (i) a clade consisting of *L. monocytogenes*, *L. marthii*, *L. innocua *and *L. welshimeri*, and (ii) a clade consisting of *L. ivanovii and L. seeligeri*. The existence of two main clades has been shown in several previous studies [3,8,23], however the placement of *L. welshimeri *has always been ambiguous. While some studies placed *L. welshimeri *basal in the *L. seeligeri*/*L. ivanovii *clade [3], others [8,23], like the majority of the phylogenetic reconstruction methods used here, place *L. welshimeri *basal in the *L. monocytogenes*/*L. marthii*/*L. innocua *clade. A likely explanation for this ambiguous phylogenetic placement is the "long branch attraction effect" [79] as *L. welshimeri *is placed on a long branch and seems to have branched off of the MRCA of the *L. monocytogenes*/*L. marthii*/*L. innocua *clade relatively early during the evolutionary of *Listeria *sensu stricto. As likelihood-based methods are less prone to long branch attraction [79], placement, by these methods, of *L. welshimeri *in the *L. monocytogenes*/*L. marthii*/*L. innocua *clade suggests that this placement is correct.

Our data also seem to support a hypothesis that the most recent common ancestor (MRCA) of *Listeria *possessed not only the *prfA *virulence cluster as indicated before [3], but also many internalins including A, B and C, which are essential for host invasion [20] and cell-to-cell spread [21]. While a few studies [19] have previously explored the evolution of internalin multigene family, including one study [19] that proposed presence of *inlB *in the MRCA of *Listeria *(except *L. grayii*), our analysis allowed for identification of 16 internalin genes that, like *inlB*, were likely present in the MRCA of *Listeria *sensu stricto.

Based on a Bayesian molecular clock analysis that used 100 genes of the *Listeria *core genome places, we propose that the MRCA of the genus *Listeria *(except *L. grayii*) can be dated to about 40 to 60 mya, similar to the date has been inferred for the most recent ancestor of *S. enterica *and *S. bongori *[80]. A plausible hypothesis for emergence of these pathogens during this time period is that a major mammalian radiation during this same epoch [81] provided strains of *Listeria *and *Salmonella *that were able to colonize mammalian hosts with a selective advantage over less adapted or environmental strains.

### Loss of virulence associated genes is a recurrent evolutionary pattern in *Listeria*

While a number of studies have reported that gene loss and genome reduction are general patterns in the evolutionary transition from facultative pathogenic lifestyles to obligate pathogenic lifestyles in bacteria [82], our data suggest that gene loss events in multiple genomic regions and lineages coincided with multiple evolutionary transitions of *Listeria *from a facultative pathogenic lifestyle to an obligate saprotrophic lifestyle. The switch from a facultative pathogen to obligate saprotrophic clades seems to have occurred at least four times during the evolutionary history of *Listeria *sensu stricto, including (i) during the speciation event leading to *L. seeligeri*, which coincided with the loss of the *inlAB *operon and *inlC*, but not the *prfA *cluster, (ii) the speciation event leading to *L. welshimeri*, which coincided with loss of the *prfA *cluster, the *inlAB *operon and *inlC*, (iii) the speciation event leading to *L. innocua*, which coincided with the loss of *inlB *and *inlC *and (iv) the speciation event leading to *L. marthii*, which coincided with the loss of the *prfA *cluster, the *inlAB *operon and *inlC*. Secondary losses of additional virulence-associated genes occurred in the non-hemolytic *L. seeligeri*, which lost the *prfA *cluster, and non-hemolytic *L. innocua *strains, which lost the *prfA *cluster as well as *inlA*.

Despite the observation that loss of virulence genes appears to be a key event in the evolution of *Listeria *species, several apparently avirulent *Listeria *strains (hemolytic *L. seeligeri *strains and hemolytic *L. innocua *strains) have strongly conserved, and in most cases functional, homologues of key *L. monocytogenes *virulence genes in their genomes. For example, previous studies [83] demonstrated some functionality of different *L. seeligeri *virulence factors and our data suggest that the homologue of internalin A in the hemolytic *L. innocua *strain supports the ability to invade human intestinal epithelial cells (even though future experiments with an isogenic *inlA *mutant will be required to confirm this). One hypothesis is that the virulence genes in *Listeria *play a role in the survival of and defense against predation by protists, however this hypothesis is not supported by a recent study that demonstrates that *L. monocytogenes *does not survive ingestion by the amoeba *Acanthamoeba polyphaga *[84].

## Conclusions

In order to gain an improved understanding of genome evolution in members of the genus *Listeria*, with a particular attention to the evolution of virulence, we generated draft genomes for seven *Listeria *strains focusing on species for which genome sequences were not previously available and atypical strains of species for which genome sequences were available (i.e., *L. monocytogenes *lineage IIIC and hemolytic *L. innocua*). Analysis of 13 genome sequences representing six *Listeria *species (including the 7 genome sequences obtained here and 6 previously reported genome sequences) suggests that (i) the genus *Listeria *possesses an open pan-genome with limited ongoing introduction of new genetic material, (ii) modern pathogenic and non-pathogenic *Listeria *species originated, approx. 40-60 mya, from a common ancestor that contained the *prfA *cluster and at least 16 internalin genes, and (iii) gene loss events played a key role in the evolution of *Listeria*. While diversification over this time period yielded a number of species-like clades in the genus *Listeria*, many of these putative species include clades or strains with atypical virulence characteristics and gene profiles. This information will be critical for the development of genetic and genomic criteria for pathogenic strains, including development of assays that specifically target pathogenic *Listeria *strains regardless of species classification.

## Competing interests

Life Technologies Corporation partially funded this study by providing sequencing reagents and instruments, and by compensating its employees (CAC, PV, LD, MB, OP, and MRF), who participated in study design, data collection and analysis, decision to publish, and preparation of the manuscript. Life Technologies Corporation also financially compensated HDB and MW for travel expenses made for a visit to Foster City.

## Authors' contributions

HDB, CAC, MW, PV, OP and MRF conceived the study. PV, LD, and MB performed the genome sequencing. HDB, CAC and PV performed the genome sequence analysis. RHO helped with the genome analysis and the SNP verification. VF performed the invasion assays. HDB, CAC, and MW wrote the paper. All authors read and approved the final manuscript.

## Supplementary Material

Additional file 1 **Primer sequences of primers used to confirm absence of specific virulence associated genes**. Click here for file

Additional file 2 **Excel file SNP differences between *L. monocytogenes *F2365 and its duplicate FSL R2-574 and results of Sanger sequencing SNP confirmation**. Click here for file

Additional file 3 **Excel file containing presence/absence data of genes in the *Listeria *pan-genome**. Click here for file

Additional file 4 **PDF file containing a graphic comparison of the inlAB region**. Click here for file

Additional file 5 **PDF file containing a graphic comparison of internalin GHE region**. Gray arrows indicate conserved genes adjacent to the region. Click here for file

Additional file 6 PDF file containing a graphic comparison of the *prfA *cluster region Click here for file

Additional file 7 **Table in MS Excel with presence or absence of 78 virulence associated genes in the genomes of the genus *Listeria***. Click here for file

Additional file 8 **PDF-file with gene trees based on internalin genes**. Click here for file
