# Supplementary material for: Comparative genomics of the bacterial genus Listeria: Genome evolution is characterized by limited gene acquisition and limited gene loss
Source: BMC Genomics. 2010 Dec 2;11:688. doi: 10.1186/1471-2164-11-688 (PMC3019230; doi:10.1186/1471-2164-11-688)
Supplement: Additional file 1 — Primer sequences of primers used to confirm absence of specific virulence associated genes. [file 1471-2164-11-688-S1.PDF]

Additional file 1. Primer sequences of primers used to confirm absence of specific virulence associated genes

region/isolate	primers	result
**inlAB**		
L. seeligeri		
FSL N1-067	LseeligeriinlABF TGCGTCCGCCTTATGGTGCA	Obtained sequence of 424 bp of intergenic region; absence of inlAB region confirmed
	LseeligeriinlABR GCGGCGACTACTTTTCGCGG	
FSL S4-171	same as FSL N1-067	Obtained sequence of 424 bp of intergenic region; absence of inlAB region confirmed
L. innocua		
FSL S4-378	S4378inlABpart1F GGCGTTGGGCGATAACAACCCT	Obtained 1520 bp of sequence: absence of inlA and inlB confirmed
	S4378inlABpart1R ACGAGACTCGACCTCTTTTGGCA	
	S4378inlABpart2F CCAAGCATTCGCTACATGGAC	
	S4378inlABpart2R TTGTTGTTGCTCTTCAGTAGTTGC	
FSL J1-023	inlBJ1023F AGCAATAGGCACCGCAGTGGT	Obtained 794 bp of sequence adjacent to 3' end of inlA: absence of inlB confirmed
	inlBj1023R TTCATCCCGAGACTCTCCACAGTC	
L. marthii		
FSL S4-120	LmainlABF GCGGTTTACACACTGACGCTCG	Obtained 503 bp of intergenic region: absence of inlA and inlB confirmed
	LmainlABR ACAGCGCCATGATGATCCCTACA	
**inlC**		
L. seeligeri		
FSL N1-067	N1067inlCpart2F AGTCGCGCAAGTAACGCGA	PCR product ca 3.5 kb; sequenced by primer walking: absence of inlC confirmed
	N1067inlCpart2R ACAGTGTTGGTGTGGAACGTACTTT	
FSL S4-171	inlCs4171F TTCCATACGGTGAAGCTGTGGAGT	Obtained 485 bp of sequence: absence of inlC confirmed
	inlCs4171R TCGCGGAGCTGGAATCAGCG	
L. innocua		
FSL S4-378	S4378inlCF TGGGGTTTTTAGTGTCAAGGCGAAT	Obtained 867 bp of sequence around gap in inlC region: absence of inlC confirmed
	S4378inlCR TGGCAGCTCCTTTGAAGTCACAGA	
FSL J1-023	J1023inlCF AGGCCGGTAACTTCTTGGGGTGT	Obtained 843 bp of sequence around gap in putative inlC region: absence of inlC confrimed
	J1023inlCR CGACTTATCAAGTTGCGGGCTATGA	
L. marthii		
FSL S4-120	LmarthiiinlC1F TGAGCTCCCGCCTAAAGTGGAGT	Closes gap in inlC region: absence of inlC confirmed
	LmarthiiinlC1F TGGCTAGTATTCTGTGCTTGGTCCT	
	LmarthiiinlC2F AGGACCAAGCACAGAATACTAGCCA	Closes gap in inlC region: absence of inlC confirmed
	LmarthiiinlC2R ACCTACGTGGTAAAGCGGCTCG	
**All species**	inlCdegF TCCAGATYCHGGYCTAGCGA	These primers were used to confirm the presence/absence of inlC in all species by PCR
	inlCdegR TGGTTYRTTYACACATTTYTGACCA	
**prfA cluster**		
L. seeligeri		
FSL N1-067	not done: *prfA* cluster present in genome sequence	
FSL S4-171	S4171prfAF CGGCACTTTGCTTAGGCCTCTGG	Obtained 686 bp of sequence, closes gaps in prfA region and confirms absence of prfA cluster
	S4171prfAR TCCAGAAACAAGTTCGTCTTCGCA	
L. innocua		
FSL S4-378	prfAS4378F GCGGTTATGTTAGTAGCGTGTGGGG	Obtained 219 bp of sequence, closes gap in prfA region and confirms absence of prfA cluster
	prfAS4378R TCCGGTTGATGCCATACCAGGT	
FSL J1-023	not done: *prfA* cluster present in genome sequence	
L. marthii		
FSL S4-120		Not done: absence of *prfA* cluster was previously confirmed*

* Graves et al. 2010. Listeria marthii sp. nov., isolated from the natural environment, Finger Lakes National Forest. IJSEM vol. 60 (Pt 6) pp. 1280-8.
