# Supplementary material for: Comparative genomics of the bacterial genus Listeria: Genome evolution is characterized by limited gene acquisition and limited gene loss
Source: BMC Genomics. 2010 Dec 2;11:688. doi: 10.1186/1471-2164-11-688 (PMC3019230; doi:10.1186/1471-2164-11-688)
Supplement: Additional file 4 — PDF file containing a graphic comparison of the inlAB region. [file 1471-2164-11-688-S4.PDF]

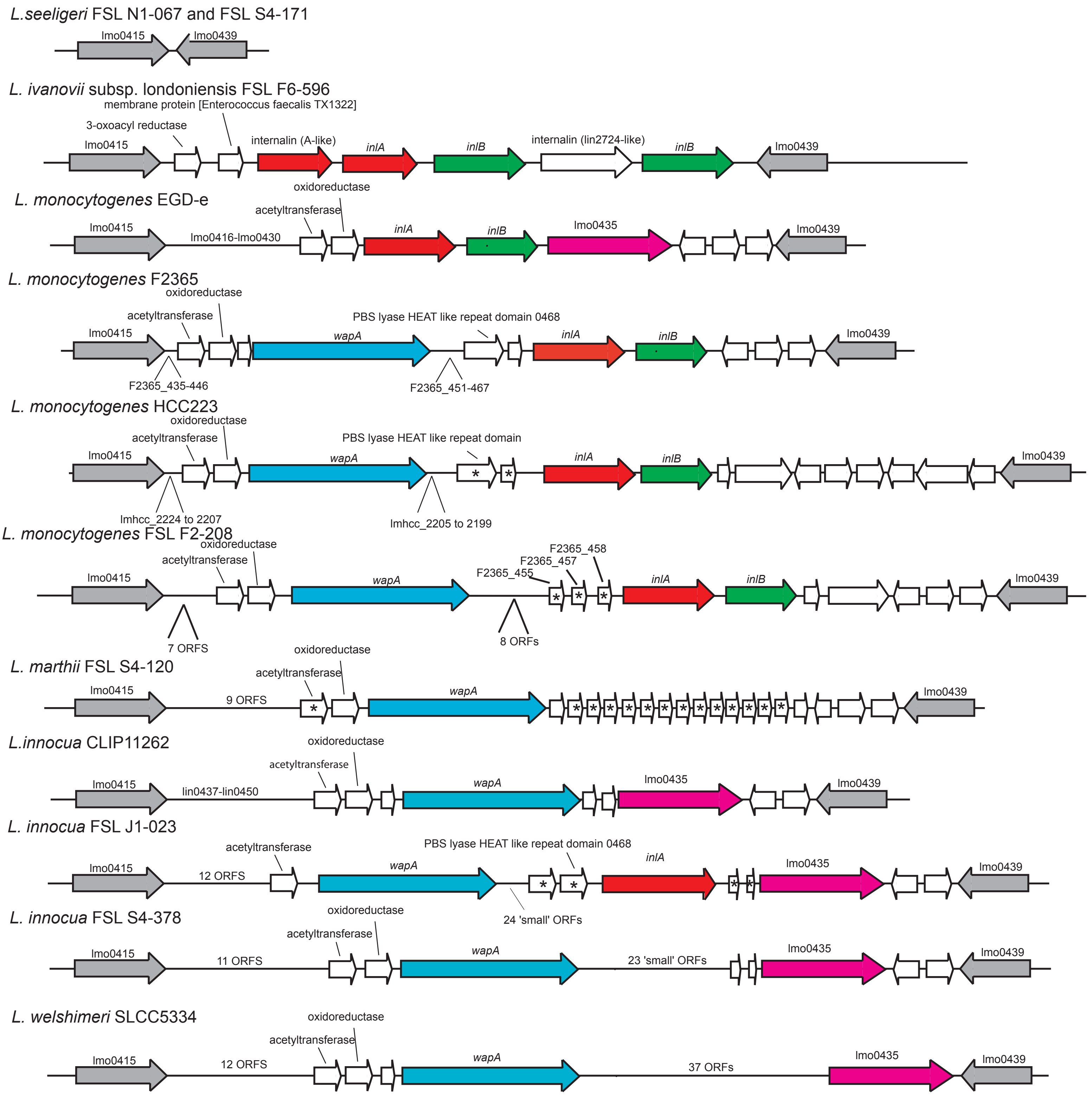

Additional file 4. Comparison of internalin AB region. Schematic representation of the internalin AB region. Gray arrows indicate conserved genes adjacent to the region; red arrows indicate inlA; green arrows indicate inlB; blue arrows indicate homologues of wapA, which encodes a wall associated protein; and magenta arrows indicate homologes of lmo435, which encodes a putative peptidoglycan bound protein. White arrows indicate ORFs that are found adjacent to surface proteins. ORFs marked with an asterisk have been identified as putative alien genes introduced in the genome by HGT.
