# Supplementary material for: Comparative genomics of the bacterial genus Listeria: Genome evolution is characterized by limited gene acquisition and limited gene loss
Source: BMC Genomics. 2010 Dec 2;11:688. doi: 10.1186/1471-2164-11-688 (PMC3019230; doi:10.1186/1471-2164-11-688)

Additional file 5. Schematic representation of the internalin GE region. Gray arrows indicate conserved genes adjacent to the region.

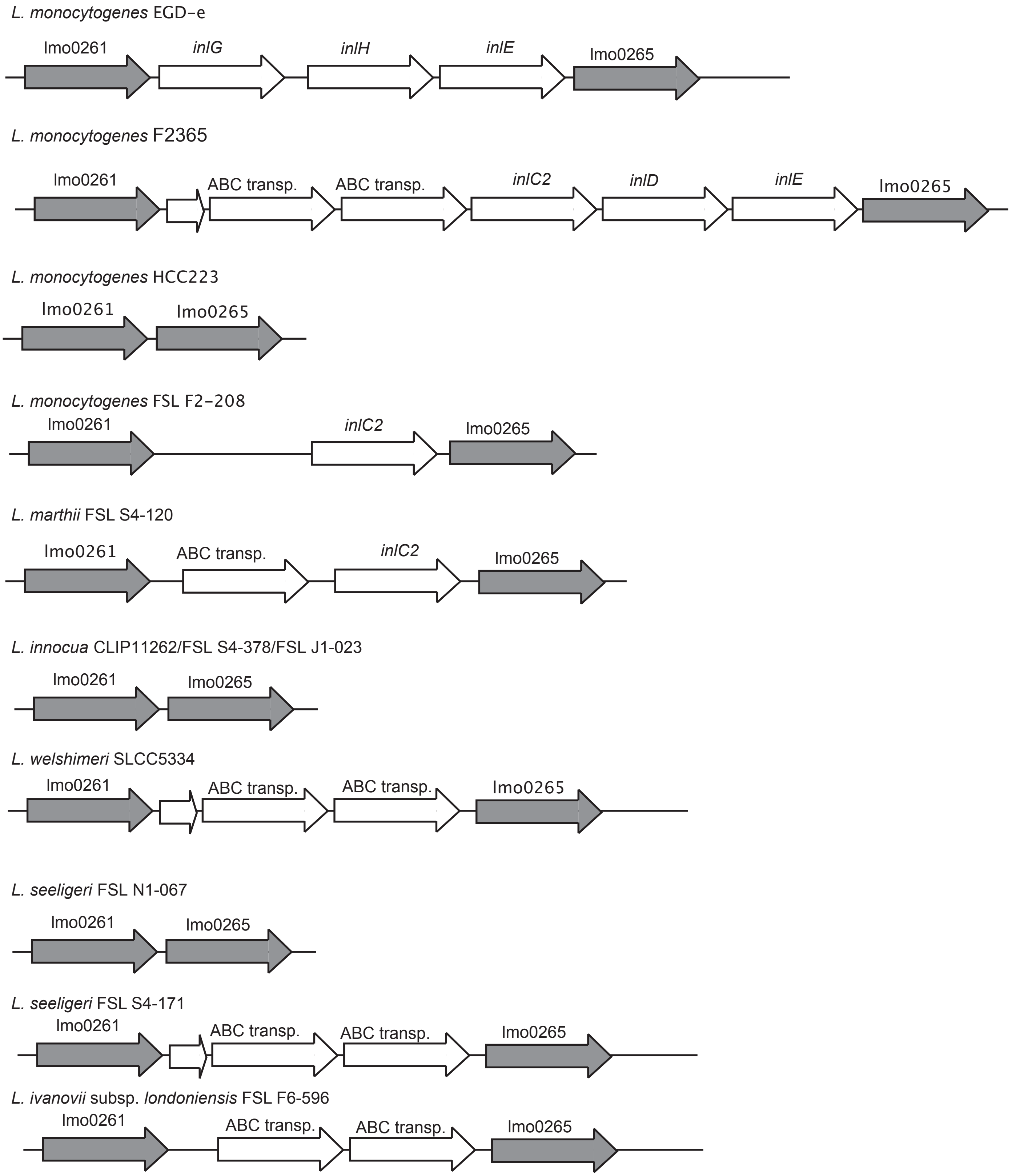

Supplement: Additional file 5 — PDF file containing a graphic comparison of internalin GHE region. Gray arrows indicate conserved genes adjacent to the region. [file 1471-2164-11-688-S5.PDF]
