# Supplementary material for: Comparative genomics of the bacterial genus Listeria: Genome evolution is characterized by limited gene acquisition and limited gene loss
Source: BMC Genomics. 2010 Dec 2;11:688. doi: 10.1186/1471-2164-11-688 (PMC3019230; doi:10.1186/1471-2164-11-688)
Supplement: Additional file 6 — PDF file containing a graphic comparison of the prfA cluster region [file 1471-2164-11-688-S6.PDF]

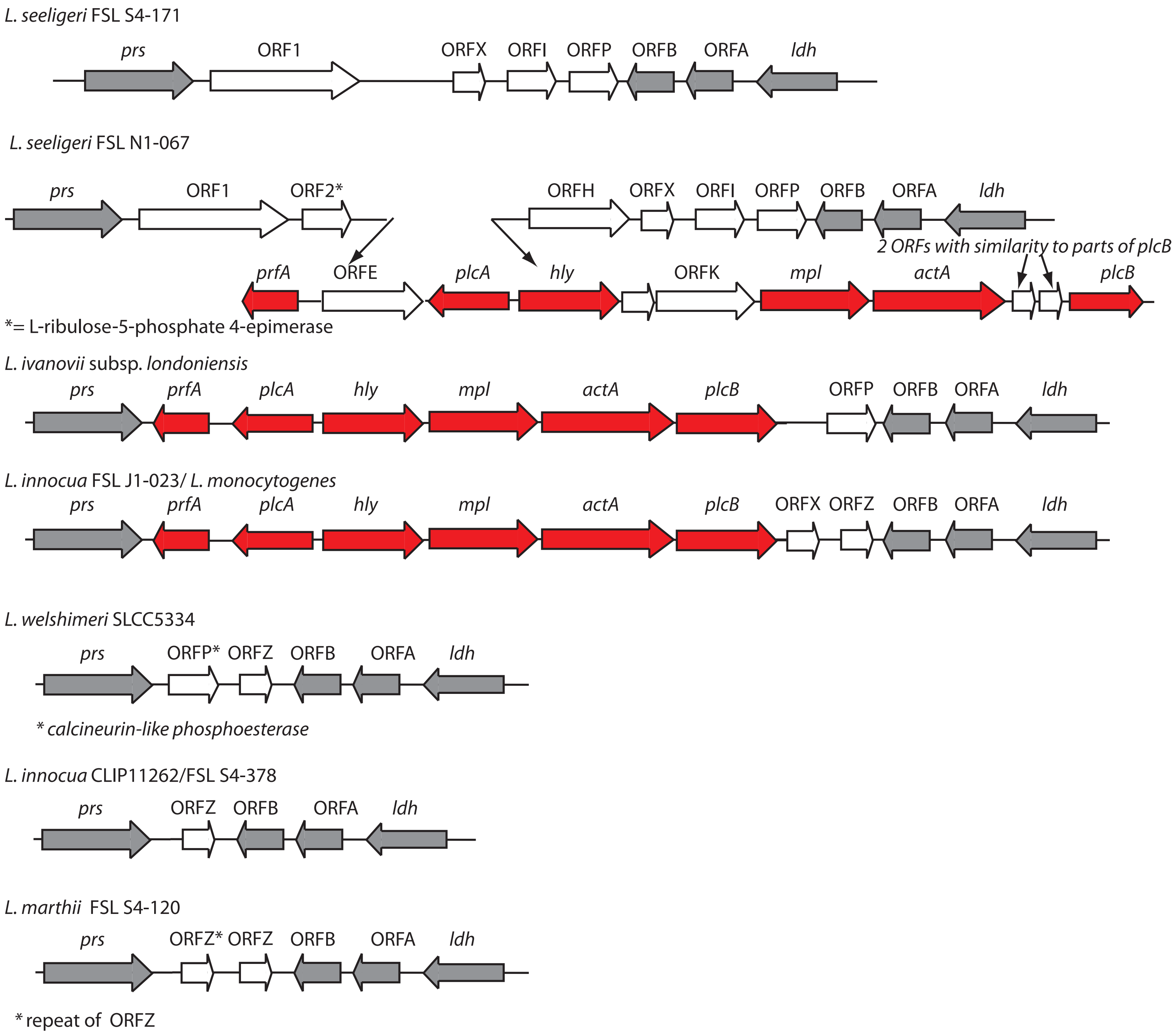


Additional file 6. Comparison of the prfA cluster region. Schematic representation of the genomic region harboring the prfA cluster. Gray arrows indicate conserved genes adjacent to the prfA cluster (prs (encoding phosphoribosyl pyrophosphate synthetase) on the left, ldh (encoding L-lactate dehydrogenase) on the right); red arrows indicate virulence associated genes in the prfA cluster.
