# Supplementary material for: Comparative genomics of the bacterial genus Listeria: Genome evolution is characterized by limited gene acquisition and limited gene loss
Source: BMC Genomics. 2010 Dec 2;11:688. doi: 10.1186/1471-2164-11-688 (PMC3019230; doi:10.1186/1471-2164-11-688)

Additional file 8. Overall internalin gene phylogeny (A) and (B) individual phylogenies of the internalin clusters found in both the *L. monocytogenes* / *L. innocua*/*L. marthii* and the *L. ivanovii*/*L. seeligeri* clade. Pages two and three show the individual phylogenies for 16 clusters that contain representatives of both main clades, indicating presence of the gene in the common ancestor. Branches with an asterisk have a posterior probability >0.95. LM_lmo = *L. monocytogenes* EGD-e, LM_f2365 = *L. monocytogenes* F2365, LM_HCC = *L. monocytogenes* HCC23, LM_FSL_F2-208 = *L. monocytogenes* FSL F2-208, LI_lin = *L. innocua* CLIP11262, LI_FSL_S4-378 = *L. innocua* FSL S4-378, LI_FSL_J1-023 = *L. innocua* FSL J1-023, LMa_FSL_S4-120 = *L. marthii* FSL S4-120, LW_lwe = *L. welshimeri* SLCC5334, LS_FSL_S4-171 = *L. seeligeri* FSL S4-171, LS_FSL_N1-067 = *L. seeligeri* FSL N1-067, and LIV_FSL F6-596 = *L. ivanovii* subsp. *londonienesis.* MrBayes version 3.12 was used to infer posterior probabilities for the individual branches; posterior probabilities were based on 700,000 post burn-in generations of a 1,000,000 generation run.

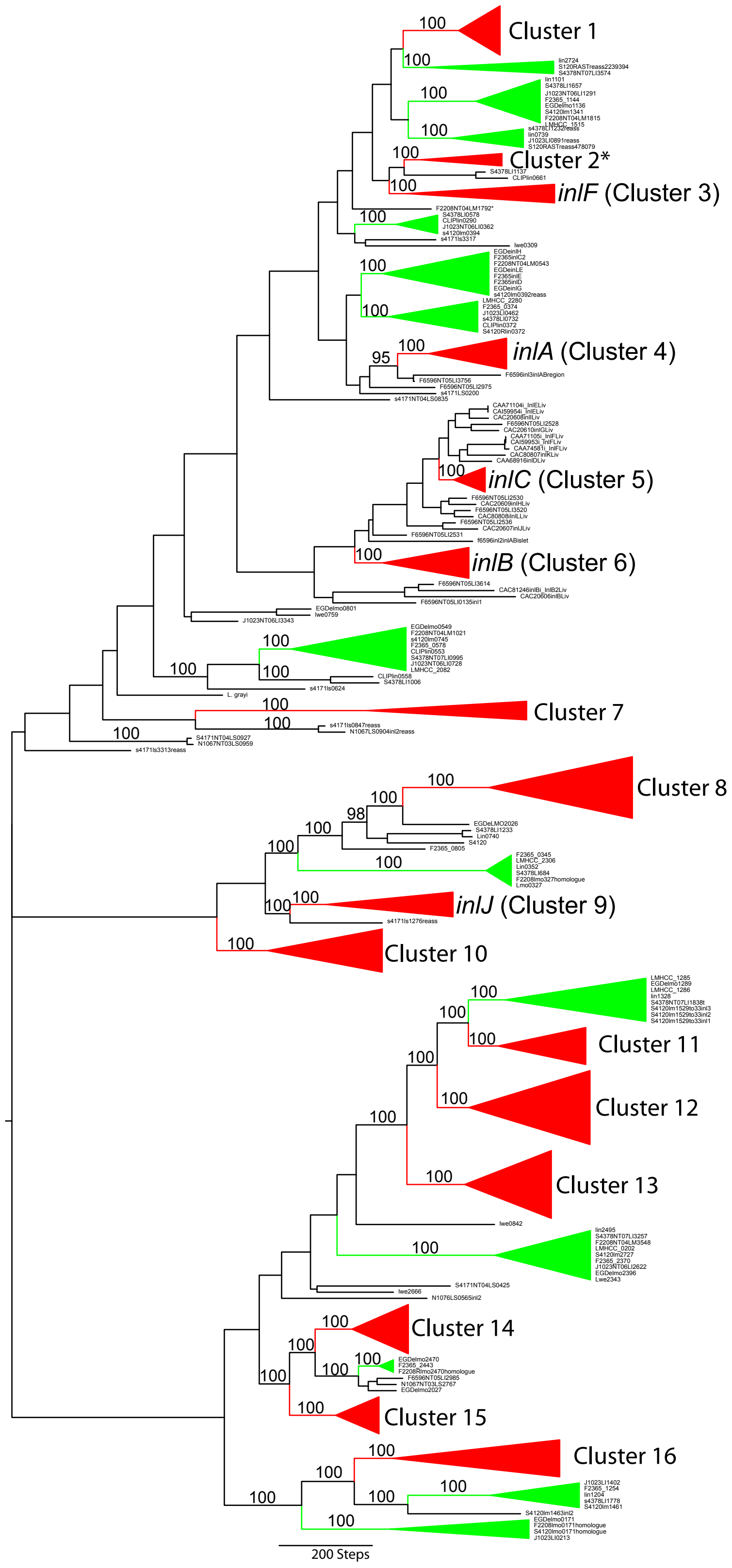

Cluster 1:

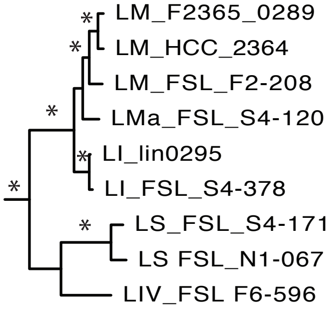

Cluster 2:

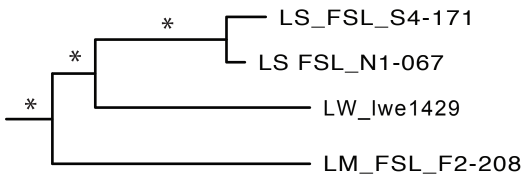

inlF (Cluster 3)

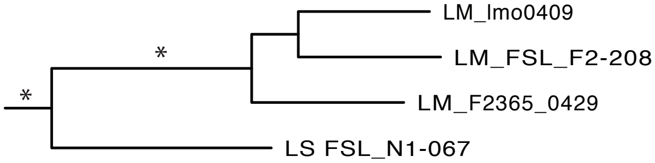

inlA (Cluster 4)

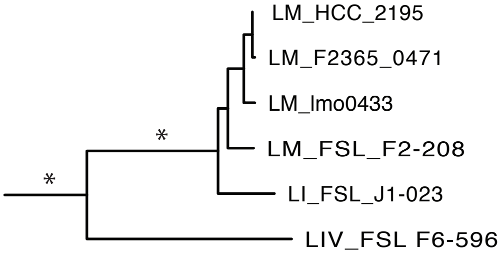

inlC (Cluster 5)

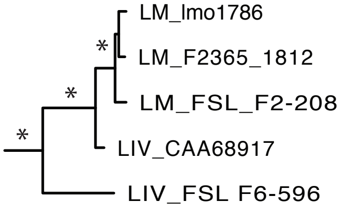

inlB (Cluster 6)

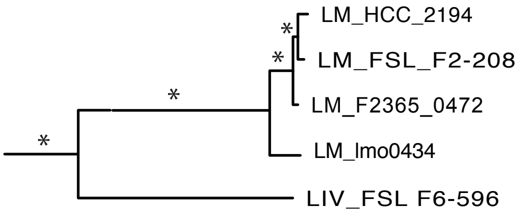

Cluster 7

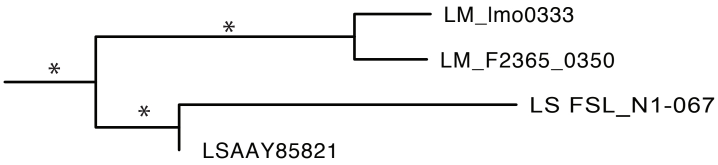

Cluster 8

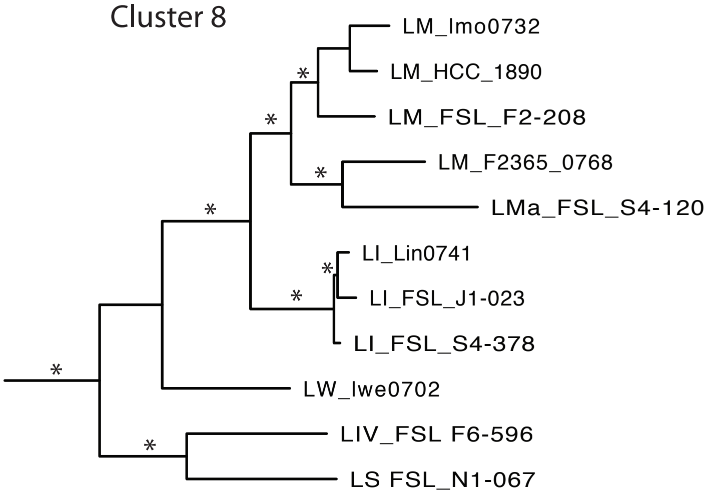

inlJ (Cluster 9)

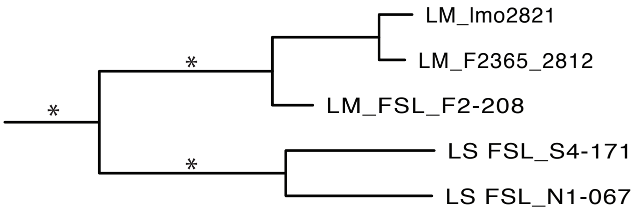

Cluster 10

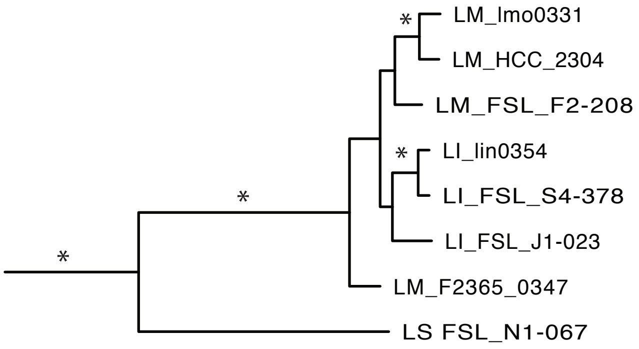

Cluster 11

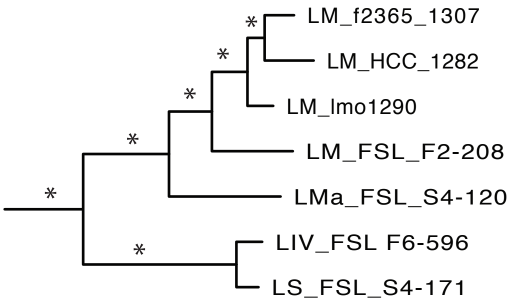

Cluster 12

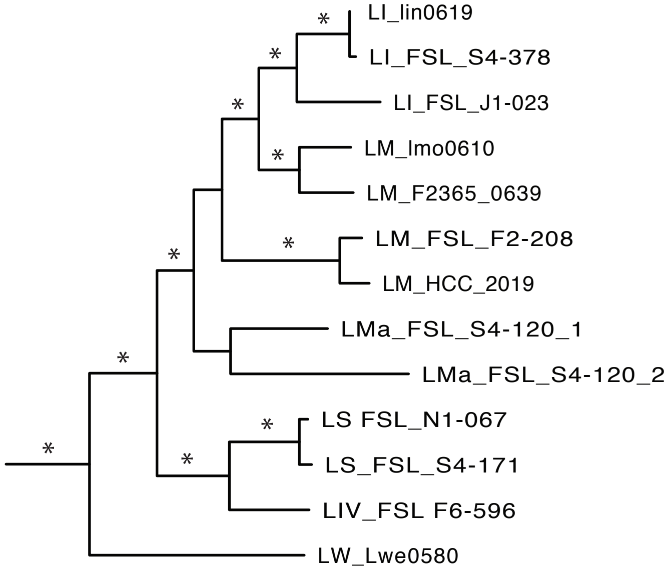

Cluster 13

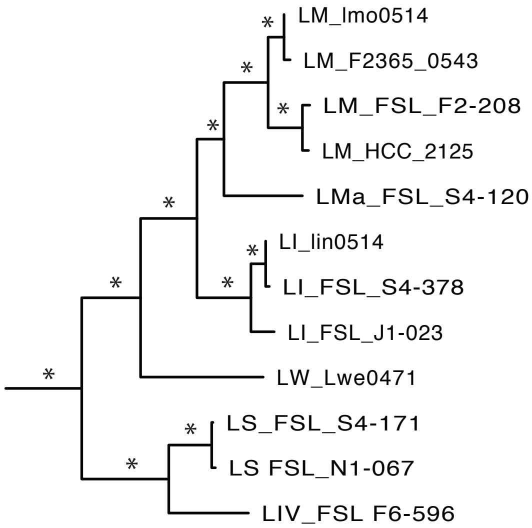

Cluster 14

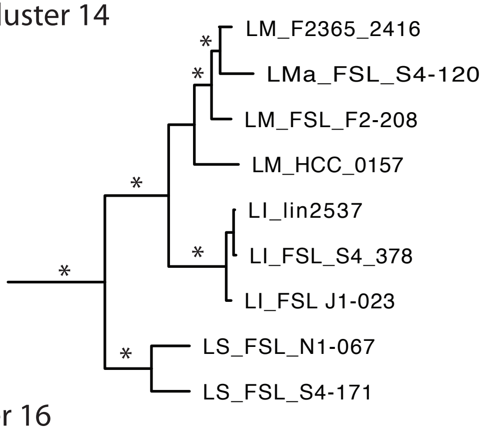

Cluster 15

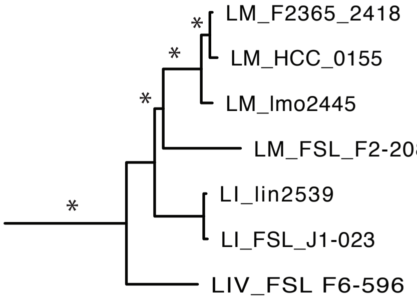

Cluster 16

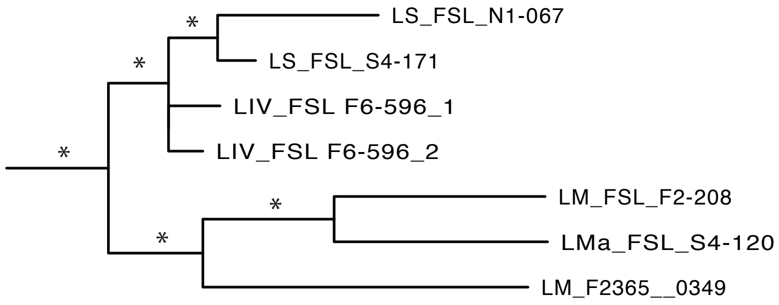

Supplement: Additional file 8 — PDF-file with gene trees based on internalin genes. [file 1471-2164-11-688-S8.PDF]
